# Whole-Organism Developmental Expression Profiling Identifies RAB-28 as a Novel Ciliary GTPase Associated with the BBSome and Intraflagellar Transport

**DOI:** 10.1371/journal.pgen.1006469

**Published:** 2016-12-08

**Authors:** Victor L. Jensen, Stephen Carter, Anna A. W. M. Sanders, Chunmei Li, Julie Kennedy, Tiffany A. Timbers, Jerry Cai, Noemie Scheidel, Breandán N. Kennedy, Ryan D. Morin, Michel R. Leroux, Oliver E. Blacque

**Affiliations:** 1 Department of Molecular Biology and Biochemistry, and Centre for Cell Biology, Development and Disease, Simon Fraser University, Burnaby, Canada; 2 School of Biomolecular and Biomedical Science, University College Dublin, Belfield, Dublin, Ireland; Karolinska Institute, SWEDEN

## Abstract

Primary cilia are specialised sensory and developmental signalling devices extending from the surface of most eukaryotic cells. Defects in these organelles cause inherited human disorders (ciliopathies) such as retinitis pigmentosa and Bardet-Biedl syndrome (BBS), frequently affecting many physiological and developmental processes across multiple organs. Cilium formation, maintenance and function depend on intracellular transport systems such as intraflagellar transport (IFT), which is driven by kinesin-2 and IFT-dynein motors and regulated by the Bardet-Biedl syndrome (BBS) cargo-adaptor protein complex, or BBSome. To identify new cilium-associated genes, we employed the nematode *C*. *elegans*, where ciliogenesis occurs within a short timespan during late embryogenesis when most sensory neurons differentiate. Using whole-organism RNA-Seq libraries, we discovered a signature expression profile highly enriched for transcripts of known ciliary proteins, including FAM-161 (FAM161A orthologue), CCDC-104 (CCDC104), and RPI-1 (RP1/RP1L1), which we confirm are cilium-localised in worms. From a list of 185 candidate ciliary genes, we uncover orthologues of human MAP9, YAP, CCDC149, and RAB28 as conserved cilium-associated components. Further analyses of *C*. *elegans* RAB-28, recently associated with autosomal-recessive cone-rod dystrophy, reveal that this small GTPase is exclusively expressed in ciliated neurons where it dynamically associates with IFT trains. Whereas inactive GDP-bound RAB-28 displays no IFT movement and diffuse localisation, GTP-bound (activated) RAB-28 concentrates at the periciliary membrane in a BBSome-dependent manner and undergoes bidirectional IFT. Functional analyses reveal that whilst cilium structure, sensory function and IFT are seemingly normal in a *rab-28* null allele, overexpression of predicted GDP or GTP locked variants of RAB-28 perturbs cilium and sensory pore morphogenesis and function. Collectively, our findings present a new approach for identifying ciliary proteins, and unveil RAB28, a GTPase most closely related to the BBS protein RABL4/IFT27, as an IFT-associated cargo with BBSome-dependent cell autonomous and non-autonomous functions at the ciliary base.

## Author Summary

Ciliopathies are genetic disorders that arise from loss or mutation of genes that encode proteins which play roles in the biology of cilia, organelles found on most of the cells in the human body. Ciliopathy-associated ailments include–but are not limited to–kidney dysfunction, blindness, skeletal abnormalities, as well as brain disorders. Although a great number of cilium-targeted proteins are known, it is thought that a large proportion remain unidentified. Here, we use a developmental gene expression series to discover novel cilia genes in the nematode *Caenorhabditis elegans*. We present several cilium-localised proteins resulting from our analysis, including RAB-28, a GTPase previously implicated in the degenerative eye disease known as cone-rod dystrophy. Through live videomicroscopy, we show that RAB-28 undergoes bidirectional transport within the cilium. A RAB-28 inactivating mutation results in loss of transport, while an activating mutation results in stronger localisation at the ciliary base and robust transport, although overexpression results in a variety of cilia-related defects. Both the wild type and activating mutant proteins require the Bardet-Biedl Syndrome-related complex of proteins for their transport, linking RAB-28 to an established ciliary transport machinery.

## Introduction

The cilium is a conserved organelle, inferred to have existed in the last eukaryotic common ancestor (LECA) and now present in most extant protists, as well as all multicellular animals. Motile cilia generate cell movement or fluid flow, whereas non-motile (primary) cilia have evolved as specialised ‘antennae’ that capture extracellular sensory cues and orchestrate extrinsic signal transduction pathways linked to development (*e*.*g*., Sonic hedgehog) [1,2]. Cilium dysfunction in humans is associated with a growing number of so-called ciliopathies that affect virtually all physiological and developmental functions [3]. For example, Bardet-Biedl syndrome (BBS) includes retinal degeneration, cystic kidneys, obesity and skeletal anomalies (polydactyly) as primary ailments [4].

Cilia are subdivided into distinct subcompartments, each with unique structural and functional features, as well as molecular compositions [5]. The canonical cilium of 9 doublet microtubules (MTs) extends from a mother centriole-derived basal body, which connects via distal appendages (transition fibers) to the plasma membrane. The proximal-most 0.2–1.0 μm of the axoneme, called the transition zone, functions in early ciliogenesis, and together with basal body structures provides a permeability barrier that separates the ciliary cytosol and membrane from the cell body [6–8]. Additional subregions include the inversin and distal tip compartments, as well as the ciliary pocket, which is a depression of the periciliary membrane where the basal body is rooted [5]. Many ciliopathy proteins and associated complexes localise to particular ciliary subcompartments, where they conduct subdomain-specific functions [5,9].

Cilia rely on various intracellular transport systems to sort and deliver the protein cargo required for cilium formation, maintenance and function [10]. The best understood is intraflagellar transport (IFT), which consists of large macro-molecular assemblies that move bidirectionally between the ciliary base and tip, driven by kinesin-2 anterograde (base to tip) and IFT-dynein retrograde (tip to base) motors [11–13]. Associated with the motors—and essential for IFT—are the IFT-A and IFT-B complexes, which likely serve as cargo adaptors [11]. The IFT-associated BBS complex (BBSome) also tethers ciliary cargo and regulates the coupling of IFT-A and IFT-B complexes [14]. Also important are membrane trafficking pathways that regulate vesicle formation and transport between post-Golgi sorting stations and the periciliary membrane, as well as endocytic retrieval and recycling events at the periciliary membrane and within the ciliary pocket [15–19]. Various IFT and ciliary membrane trafficking regulators have been identified, including small GTPases of the RAB, ARF and ARL families, that function during early cilium formation as well as transport events post-ciliogenesis [10,20,21].

Given the multifaceted roles of cilia, together with its prevalent disease association, there have been major efforts to identify the ‘ciliome’, or complete molecular parts list of cilia, using a wide range of cell types and organisms [22–24]. Approaches have included comparative genomics of ciliated *versus* non-ciliated species [25], identification of binding sites for the ciliogenic transcription factors DAF-19/RFX or FOXJ1 [26–31], expression analyses involving microarray, serial analysis of gene expression (SAGE) and RNA-Seq [25,28,32–37], as well as proteomics [38–41]. Data from such studies are compiled in the online ciliary database, Cildb [23,24]. Whilst the studies have contributed immensely to understanding cilia biology, each approach has limitations and additional ciliary components almost certainly remain unidentified.

*C*. *elegans* represents a powerful genetic model for investigating cilium formation and function [22]. Hermaphrodite worms possess 60 ciliated cells (of 960 total), all of which are sensory neurons. The non-motile sensory cilia extend from the dendritic tips and many are contained within bilateral chemo- and thermo-sensory cuticular organs, supported by glial cell (sheath and socket) processes that establish environmentally exposed channels [42–44]. *C*. *elegans* cilium morphologies range from the canonical rod-like to forked, multi-branched and membrane-expanded structures [22]. Worm cilia also possess ultrastructural features conserved in vertebrate/mammalian cilia; for example, amphid (head) and phasmid (tail) channel cilia possess long A-tubule extensions that establish a proximal axonemal region or ‘middle segment’ of 9 outer doublet MTs and a ‘distal segment’ of 9 outer singlet MTs [22]. Because many ciliary genes and pathways are conserved in nematodes, and complete loss of cilia is non-lethal [26], *C*. *elegans* has been a leading metazoan model for discovering new ciliary genes and uncovering new insight into ciliary transport, function and disease mechanisms.

In this study, we identified a unique expression profile for ciliary genes using a series of RNA-Seq libraries generated specifically to improve annotation of the transcriptome [45–47]. We confirmed that our clustering analysis identifies known ciliary proteins, including several not previously studied in *C*. *elegans*, and uncovers novel conserved ciliary proteins. One of these proteins is RAB-28, which is expressed exclusively in ciliated cells, where it associates with the periciliary membrane and behaves as an IFT cargo *via* BBSome- and nucleotide binding-dependent mechanisms. Overexpression of predicted active or inactive forms of RAB-28 leads to variant-specific ciliary and cell non-autonomous sensory pore morphogenesis defects. Together, our work provides a novel approach to finding new ciliary proteins, and uncovers a functional association between the BBSome, IFT and the orthologue of the cone-rod dystrophy protein, RAB28.

## Results

### Whole-organism developmental expression profiling reveals a cilium-specific gene expression pattern

**Fig 1:**
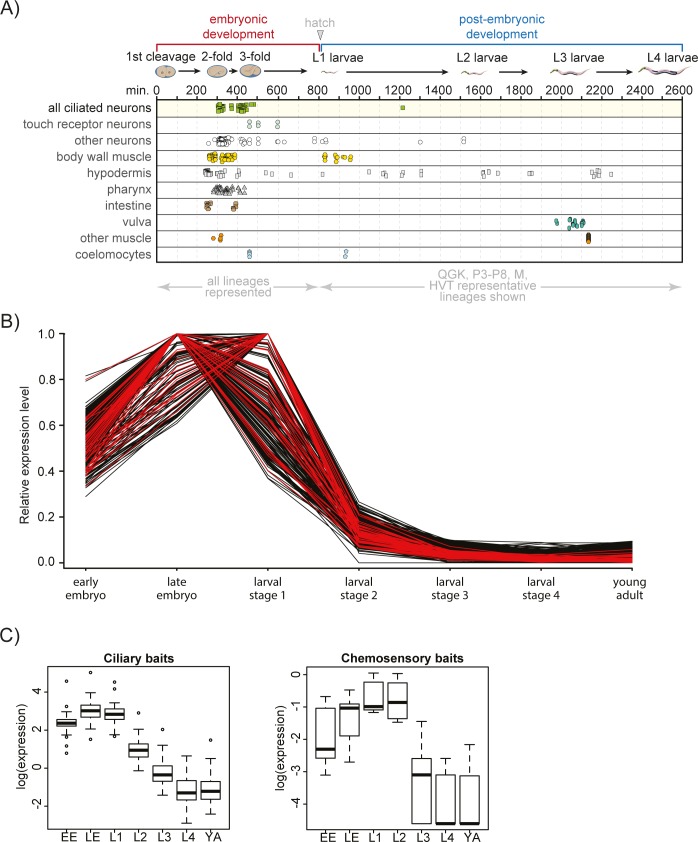
Whole-organism expression profiling identifies a signature pattern enriched for known ciliary genes. **(A)** Timeline (in minutes) of *C*. *elegans* cell births according to tissues. Small shapes in each row represent the birth of a cell of the corresponding tissue type. Embryonic development, hatching and post-embryonic development are highlighted above the x-axis with the worm stages illustrated below. Most ciliated neurons are born in late embryogenesis with ciliogenesis occurring shortly after these cells are born. Worm illustrations modified from WormAtlas (www.wormatlas.org). Q/G/K/P/H/V/T/M refer to different embryonic cell lineages (http://www.wormatlas.org/celllineages.html). **(B)** Normalised expression levels for all ‘bait’ cilium-associated genes (red) and the top 276 target genes (black, p-value < 5e-7) over the developmental series of analysed RNA-Seq libraries. Expression peaks for all genes during late embryo or larval stage 1, which matches closely the birth of the ciliated neurons shown in (A). This cilia-specific gene expression pattern shows much lower levels of expression from larval stage 2 to young adult. **(C)** Box and whisker plots of the normalised expression across the whole-organism developmental RNA-Seq libraries for the chemosensory and ciliary gene sets. Expression of the ciliary set immediately precedes that of the chemosensory set. Peak expression for ciliary genes is at the late embryo-L1 larval stage, whereas expression peaks at L1-L2 larval stages for chemosensory genes. EE; early embryo. LE; late embryo. L1; larval stage 1. L2; larval stage 2. L3; larval stage 3. L4; larval stage 4. YA; young adult.

To provide a complementary approach to ciliary gene discovery, we took advantage of the temporally-invariant birth of all *C*. *elegans* cells and tissues, including ciliated neurons, during development [48] (Fig 1A). Nearly all of the 60 ciliated neuronal cell types in *C*. *elegans* hermaphrodites are born within a discrete embryonic time period 300–450 minutes post-fertilisation, with cilium formation occurring very shortly thereafter (Fig 1A). We hypothesised that ciliogenesis genes are highly expressed during this time period and therefore distinguishable from genes required for general neuronal formation and development, which are expressed during a broader time window (Fig 1A). Using an available whole-organism developmental series of RNA-seq libraries from *C*. *elegans* [45–47], we confirmed this hypothesis: many well-characterised cilia genes are highly expressed in the early embryo, display peak expression in the late embryo and first larval stage, and show greatly reduced expression during subsequent developmental stages (Fig 1B).

Next we sought to identify novel ciliary/ciliogenic genes displaying a similar cilia-related expression pattern. Using a set of 41 well-characterised ciliary component genes as “baits”, representing the cilia-related gene expression profile during development (sheet 1 in S1 Table), we queried our ciliary transcriptome to identify other gene “preys” with similar expression profiles across the RNA-Seq libraries (Fig 1B and sheet 2 in S1 Table). Hierarchical clustering of genes based on temporal expression reveals a tight cluster of 34 of the bait genes with 151 prey genes (cluster 1), many of which are uncharacterised (S1 Fig and sheet 3 in S1 Table). We also filtered the gene list to only include those with human orthologues [49]. To validate our dataset, we determined if filtered cluster 1 is enriched for genes with >12 hits in the ciliary database Cildb [23,24] (sheets 3 and 4 in S1 Table). We found that conserved Cildb-represented genes are significantly enriched (84.5 fold) in filtered cluster 1 compared to the entire genome (p<0.0001, chi-squared test; sheet 4 in S1 Table). We further compared the cluster 1 gene set to two previous expression studies [25,50] and see significant overlap between lists (p<0.0001; sheet 4 in S1 Table).

Because our genes were identified by shared temporal gene expression, we reasoned that they may share common promoter regulatory elements beyond the previously identified X-box motif [27,28] and so we performed a motif discovery analysis on the conserved genes in cluster 1 (sheet 3 in S1 Table). Not surprisingly, we find several motifs that are significantly enriched, including matches to the GAGA factor binding site (S2 Fig). As might be expected, the 14-bp X-box was not identified by the motif elucidation program despite its enrichment in our dataset (sheet 4 in S1 Table), likely due to the number of degenerate bases in its consensus sequence and because the program identifies motifs only up to 8-bp long [26–28,51]. None of the elements we identified have been previously associated with cilia gene expression.

### Post-ciliogenesis chemoreceptor co-expression profiling

We observed that chemoreceptors (G protein-coupled receptors or GPCRs) [52], many of which are known to be cilium-localised, are largely absent from our ciliary dataset (sheet 3 in S1 Table). We hypothesised that such genes might be transcribed post-ciliogenesis. Using a statistical predictive modelling strategy similar to that used above, we identified 80 genes sharing the expression profile of *srg-36*, a cilium-localised dauer pheromone receptor required for entry into the dauer larval diapause life stage (Fig 1C and sheet 5 in S1 Table) [53]. Of the 80 genes, 27 are serpentine transmembrane receptors, representing a significant 7-fold enrichment over all such genes in the genome (p<0.0001, chi-squared test). Analysis of the upstream regulatory regions revealed that 25 of the 28 chemoreceptor genes (including *srg-36*) possess an E-box regulatory element (S3 Fig) [54]. This suggests that their similar temporal expression profiles stem from regulation by the same, or similar, transcription factors such as the E-box-binding Basic Helix-Loop-Helix bHLH transcription factor [54]. Interestingly, 4 of the predicted serpentine receptors, including *srg-36*, are known to be expressed in ciliated sensory neurons [53,55,56]. We note that the rise and fall of chemosensory/GPCR gene expression trails that of the cilia-related genes (Fig 1C), indicating that these two expression profiles of related developmental processes are temporally discrete.

### Identification of previously unstudied proteins from *C*. *elegans* that are cilium-localised: FAM-161, CCDC-104, CCDC-149, RPI-1, MAPH-9, YAP-1 and RAB-28

To confirm that our predictive expression profiling model identifies novel ciliary proteins in *C*. *elegans*, we used GFP reporters to investigate the cell and tissue expression patterns, and subcellular protein localisations, of seven previously uncharacterised *C*. *elegans* proteins from filtered cluster 1, namely FAM-161 (Y38H6C.14), CCDC-104 (Y108G3Al.3), RPI-1 (W07G1.5), RAB-28 (Y11D7A.4), CCDC-149 (F29G6.2), MAPH-9 (C34D4.1) and YAP-1 (F13E6.4). These were chosen because at the onset of this study there was little or no published evidence of ciliary associations for most of these proteins in any system. Specifically, we made transcriptional ‘promoter fusion’ reporters (endogenous gene promoter fused to GFP) for CCDC-104, CCDC-149 and RAB-28, and translational ‘protein fusion’ reporters (endogenous gene promoter + genomic exon/intron or cDNA sequence fused to GFP) for all 7 candidates. The only exception was the YAP-1 translational reporter, where the *bbs-8* gene promoter active only in ciliated cells [57] was used because of the widespread expression of YAP-1 in multiple tissues and cell types [58]. We also made a second RAB-28 translational reporter driven by the *bbs-8* gene promoter.

**Fig 2:**
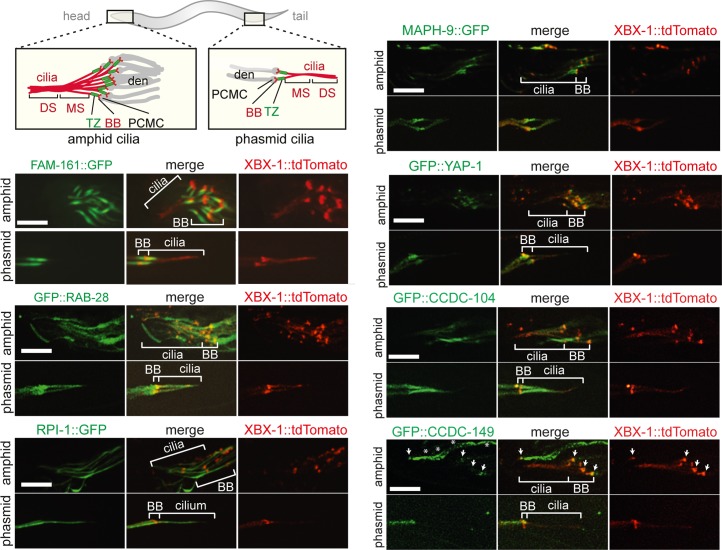
Candidate cilium-associated proteins localise to ciliary structures Representative images of amphid (head) and phasmid (tail) cilia from N2 wild type worms co-expressing GFP-tagged ‘translational’ reporters for candidate ciliary genes and tdTomato-tagged XBX-1 (IFT protein that localises to the ciliary base and axoneme). All reporters driven by the endogenous gene promoter, except for YAP-1 and RAB-28, where a *bbs-8* gene promoter sequence was used (see Fig 3A for a GFP::RAB-28 reporter driven by the endogenous promoter). GFP tags are on the N- or C-terminus, as indicated above the panels. PCMC (periciliary membrane compartment); distal dendrite (den) swelling enriched for endocytosis-associated proteins and vesicles that regulate ciliary membrane homeostasis [19]. The transition zone (TZ) extends from basal body (BB) and functions as a ciliary gate that regulates protein entry and exit to and from cilia [6]. Middle (MS) and distal (DS) segments; characterised by a circular array of 9 doublet (A and B tubules) and 9 singlet (A tubules) microtubules, respectively (not shown). Note that for GFP::CCDC-149, signals are observed at the amphid/phasmid basal bodies (arrows) and as punctae (asterisks) along the dendrite of the OLQ ciliated neuron running parallel to the amphid neurons. Scale bars; 5 μm.

**Fig 3:**
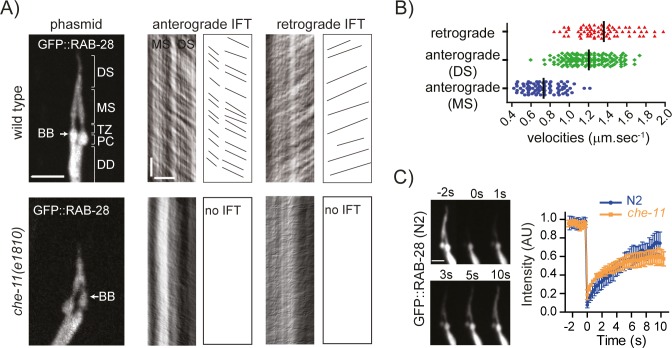
RAB-28 undergoes IFT. **(A, B)** Representative images of phasmid cilia (left) from N2 wild type and *che-11(e1810)* worms expressing GFP::RAB-28, together with corresponding kymographs and kymograph schematics derived from time-lapse imaging. Distribution plot (and mean values) in B shows kymography-determined anterograde and retrograde GFP::RAB-28 velocities from wild type worms. MS; middle segment, DS; distal segment, PC; periciliary membrane compartment, DD; distal dendrite. Scale bars; 3 μm (phasmid image; and horizontal bar on kymographs); 3 seconds (vertical bar on kymographs). **(C)** Fluorescence recovery after photobleaching (FRAP) plots for GFP::RAB-28 in the phasmid neurons of N2 and *che-11(e1810)* mutant worms. GFP::RAB-28 also undergoes free diffusion in wild-type (N2) and *che-11* IFT mutant animals. Ciliary GFP signals bleached at time 0. Intensity measurements normalised to pre-bleach levels. Curves derived from 3 separate FRAP experiments. Error bars; SEM. Images taken from a representative FRAP experiment in N2 wild type worms. s; seconds. Scale bar; 2 μm.

All reporters employing the endogenous gene promoter show enriched or almost exclusive expression within ciliated cells (S4A Fig), thus validating the predictions from our coexpression profiling. Specifically, *maph-9*, *rpi-1*, *ccdc-104*, *rab-28*, and *fam-161* are exclusively expressed in ciliated neurons, with *ccdc-149* expressed in most ciliated neurons as well as pharyngeal neurons, touch receptor neurons, and motor neurons as previously reported (Figs 2 and S4A) [59]. Furthermore, the translational reporters reveal that all 7 proteins localise at the base of, or within, cilia (Fig 2 and Fig 3A). FAM-161::GFP signals are found at the ciliary base and within the proximal part of the ciliary axoneme, including the transition zone (TZ) (Fig 2). This localisation is similar to that of the mammalian protein implicated in retinitis pigmentosa, and is consistent with the suggested role of FAM161b in cargo delivery to photoreceptor outer segments (cilia) [60,61]. Both GFP reporters for RAB-28 (driven by *bbs-8* gene or endogenous promoter), the orthologue of the small ciliary GTPase RAB28 linked to human autosomal-recessive cone-rod dystrophy [62–64], localise along the entire cilium. (Fig 2 and Fig 3A). Ciliary axoneme localisations are also observed for the GFP-tagged RPI-1, MAPH-9, YAP-1 and CCDC-104 translational reporters; in contrast, CCDC-149::GFP is absent from the ciliary axoneme, although a pool of signal is evident at the ciliary base (Fig 2). The ciliary localisation of CCDC-104::GFP is consistent with the mammalian ciliary base and axonemal localisations reported for the ARL3 interacting protein, CCDC104/BARTL1 [65]. We also found that RPI-1::GFP appears to localise to ciliary and cytoplasmic microtubules, similar to its mammalian orthologue (RP1) implicated in retinitis pigmentosa (Fig 2) [66]. Our finding of ciliary localisations for YAP-1, MAPH-9 and CCDC-149 are the first in any cellular system or organism, and is consistent with a known ciliogenesis role for mammalian YAP, and cilia-related phenotypes in MAP9 (MAPH9 orthologue)-disrupted zebrafish and dachshunds [67–70]. YAP is regulated by the ciliary protein NPHP4, and MAP9 is phosphorylated by the cilia-disassembly/centrosomal kinase PLK1 [71,72].

We also examined genes excluded from filtered cluster 1 due to a reported lack of predicted human orthologues (according to the Ortholist database; [49]). Highly ranked in unfiltered cluster 1 is an uncharacterised gene with an X-box promoter element [26], *tza-3*, which we demonstrate is expressed exclusively in ciliated neurons, and encodes a ciliary TZ protein (S4B Fig). Whilst TZA-3 is not conserved, the TZ localisation of this predicted transmembrane protein depends on the highly conserved core TZ scaffolding protein MKS-5 and “MKS module” components MKSR-1 and MKSR-2, but not the “NPHP module” protein NPHP-4 (S4B Fig). These findings are consistent with TZA-3 associating with the MKS module, which plays a role in ciliary gating [6–8]. Thus, our expression profiling approach identifies non-conserved ciliary proteins in addition to conserved ciliary proteins.

Of the novel *C*. *elegans* ciliary proteins we identified, we sought to characterise RAB-28 further for the following reasons: (1) human RAB28 is associated with a possible ciliopathy (cone-rod dystrophy [62,63]), (2) many small GTPases play essential roles in ciliogenesis and ciliary membrane trafficking pathways [10], and (3) aside from reports of RAB28 localising at the basal body [62] or the cilium [64], there is a complete absence of molecular studies on this evolutionarily conserved protein that support a functional and mechanistic link to cilia.

### RAB-28 undergoes intraflagellar transport (IFT)

We further investigated the ciliary localisation of GFP::RAB-28 using time-lapse imaging and found that this GTPase undergoes continuous, bidirectional, IFT-like movement along ciliary axonemes (S1 Movie). In contrast, the additional non-ciliary GFP::RAB-28 signals present throughout the neurons are diffuse, with no processive movement detected. In *C*. *elegans* amphid and phasmid cilia, anterograde IFT is driven by two kinesin-2 motors (kinesin-II and OSM-3) to yield distinct anterograde rates along the middle and distal segments [14,73]. Kymograph analysis of phasmid (tail) cilia confirm that ciliary GFP::RAB-28 moves at IFT-associated velocities, displaying characteristic average anterograde rates of ~0.7 μm/sec (middle segment; proximal part of axoneme) and ~1.2 μm/sec (distal segment; distal part of axoneme), and retrograde rates of ~1.4 μm/sec (along entire cilium length) (Fig 3A and 3B and S1 Movie) [73]. To examine if this ciliary trafficking is truly associated with IFT, we examined GFP::RAB-28 in worms with disrupted CHE-11 (IFT140), which is a component of IFT-A essential for IFT [28,74,75]. Although GFP::RAB-28 is observed within the truncated cilia of *che-11* mutants, we could not detect processive movement of the GFP signals, indicating that the trafficking behaviour of RAB-28 within cilia is *bona fide* IFT (Fig 3A and S1 Movie). In contrast to observations for other IFT-associated proteins in IFT deficient-worms [28,74,75], GFP::RAB-28 does not abnormally accumulate at the ciliary base or tip, or along the axoneme of these animals (Fig 3A). This lack of accumulation, despite the active transport defect, suggests that our GFP::RAB-28 reporter also freely diffuses within (and between) the ciliary and dendritic compartments, in addition to undergoing IFT. We confirmed this hypothesis using a fluorescence recovery after photobleaching assay in wild type and *che-11* mutant worms, which show that GFP::RAB-28 is highly mobile, displaying very rapid exchange kinetics between ciliary and dendritic pools (Fig 3C). Thus, in addition to active transport via IFT, GFP::RAB-28 undergoes IFT-independent free diffusion, which explains why this reporter does not abnormally accumulate within *che-11* mutant cilia.

### RAB-28 is an IFT cargo

To determine if RAB-28 is required for IFT, we examined ciliary structure, function and protein transport in worms containing a deletion (*gk1040*) in *rab-28*. The *gk1040* mutation removes the GTP-binding switch II domain and the farnesylated C-terminal CAAX motif (S5A Fig), both of which are critical for RAB protein function [76]; thus, *gk1040* is likely a severe loss-of-function or null allele. We outcrossed the *gk1040* background at least 3 times with wild type worms to remove background mutations unlinked to *gk1040*. We find that ciliated amphid and phasmid sensory neurons in *rab-28* mutant worms display a normal dye filling response, which suggests that ciliary structures are intact (short cilia usually abrogate dye uptake due to lack of environmental exposure) [77] (S5B Fig). Localisation of an IFT protein, OSM-6 (IFT52 orthologue), throughout normal-length cilia confirms this finding (S5C Fig). In addition, transmission electron microscopy (TEM) analyses reveal that ciliary ultrastructures appear normal in *rab-28* mutants (S5D Fig). Furthermore, these worms are normal for cilia-related sensory behaviours (S5E Fig), as well as cilium-dependent carbon dioxide avoidance and development (body size) (S5F Fig) [78–80]. Finally, the localisation (and movement) of BBSome (BBS-5), IFT-A/B and various ciliary membrane proteins is also grossly normal in *rab-28* mutants (S5G Fig). Thus, unlike the disruption of IFT proteins, loss of RAB-28 does not affect ciliary structure, transport, or function, at least for those proteins and cilia that were analysed. We conclude, therefore, that RAB-28 behaves more like an IFT cargo, or peripherally-associated component of IFT complexes, rather than a central component of the machinery required for bidirectional movement (IFT).

### RAB-28 association with the periciliary membrane and IFT is dependent on GTP-binding and the BBSome subunit orthologue, BBS-8

**Fig 4:**
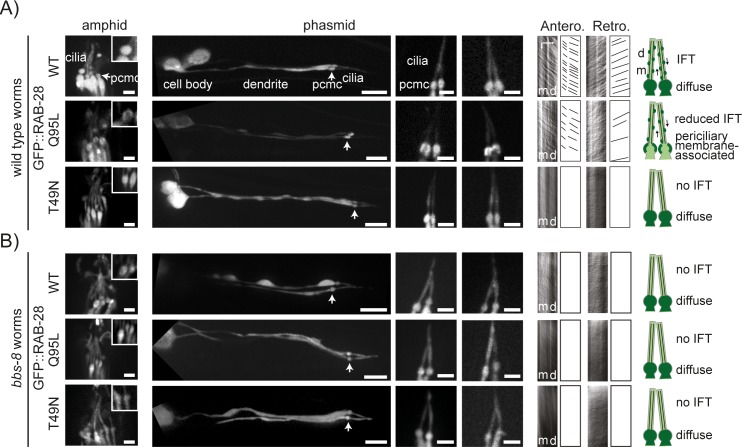
BBSome-dependent recruitment of activated RAB-28 to the periciliary membrane. **(A, B)** Representative images of amphid and phasmid cilia from N2 wild type (A) and *bbs-8(nx77)* mutant (B) worms expressing GFP-tagged RAB-28(WT), RAB-28(GDP) and RAB-28(GTP) reporters. All reporters are driven by the endogenous *rab-28* gene promoter. Kymographs and kymograph schematics derived from time-lapse imaging of GFP signals in phasmid cilia. Phenotypes summarised in cartoons. Large phasmid images are placed on black backgrounds. m; middle segment, d; distal segment. pcmc; periciliary membrane compartment (also denoted by arrow). Scale bars; 2 μm and 5 μm (low magnification phasmid images).

Next, we assessed the requirements of GDP and GTP nucleotide binding for RAB-28 localisation and transport. We made GFP-tagged constructs containing T49N or Q95L mutations in RAB-28, which are predicted to trap the GTPase in GDP (inactive) or GTP (active)-locked states, respectively [81]. These constructs were injected into wild type worms at the same concentration (5 ng/μl) as the GFP::RAB-28(WT) construct described above (Fig 3), and all appear to be expressed at similar levels. Like the RAB-28(WT) reporter, RAB-28(T49N) (hereafter termed RAB-28(GDP)) displays diffuse localisation throughout the entire neuron, including the cilium and its associated periciliary membrane compartment (PCMC) (Fig 4A). However, unlike wild type RAB-28, RAB-28(GDP) fails to undergo detectable IFT, indicating a highly reduced ability to associate with IFT trains (Fig 4A and S1 Movie). In contrast, the localisation of the RAB-28(Q95L) (hereafter termed RAB-28(GTP)) is highly enriched at the PCMC compared with the rest of the cell, forming a ‘tunnel-like’ localisation indicative of association with the periciliary membrane (Fig 4A). RAB-28(GTP) also undergoes clearly detectable IFT, albeit at an apparently reduced frequency compared to the wild type reporter (Fig 4A and S1 Movie). Notably, the localisation and IFT behaviour of all three GFP::RAB-28 reporters (WT, GDP-locked, GTP-locked) is not altered when expressed in the *rab-28(gk1040)* null background (S6 Fig), indicating that endogenous RAB-28 does not affect the distribution and transport behaviour of the RAB-28 markers. Furthermore, transgenic worms expressing GFP::RAB-28(GTP) at a very low level (injected at 0.5 ng/μl; *oqEx304*) is exclusively localised at the periciliary membrane and undergoes IFT (S7 Fig and S2 Movie), thus confirming our observations with the higher expressing marker, and indicating that the periciliary membrane is the primary site of activated RAB-28 function in nematode sensory neurons (Fig 4A). Taken together, these data show that GTP binding targets RAB-28 to the periciliary membrane and facilitates its association with IFT trains.

These observations for *C*. *elegans* RAB-28 are reminiscent of those for mammalian RAB8, whose ciliary targeting also depends on GDP-GTP exchange, via a mechanism involving RABIN8 (RAB8 guanine nucleotide exchange factor) and BBSome functions at the base of cilia [82–84]. We therefore examined the localisation and transport behaviour of all three GFP-tagged RAB-28 reporters (WT, GDP-locked and GTP-locked) in a likely null allele (*nx77*) of a *C*. *elegans* BBSome gene orthologue, *bbs-8*. The *bbs-8(nx77)* mutation does not alter the ciliary targeting and distributions of RAB-28(WT) and RAB-28(GDP) compared to a wild type background (Fig 4B). In contrast, BBS-8 loss has a striking effect on both of our RAB-28(GTP) reporters, preventing their accumulation at periciliary membranes (Fig 4B and S7 Fig). Furthermore, the IFT movement of RAB-28(WT) and RAB-28(GTP) is abolished in *bbs-8* mutant cilia (Fig 4B and S1 Movie). From these data, we conclude that the IFT-associated BBSome is required for targeting activated RAB-28 (GTP-locked) to periciliary membranes and loading onto IFT trains.

### Overexpression of constitutively active or inactive RAB-28 induces distinct cell autonomous and cell-non autonomous sensory pore defects

**Fig 5:**
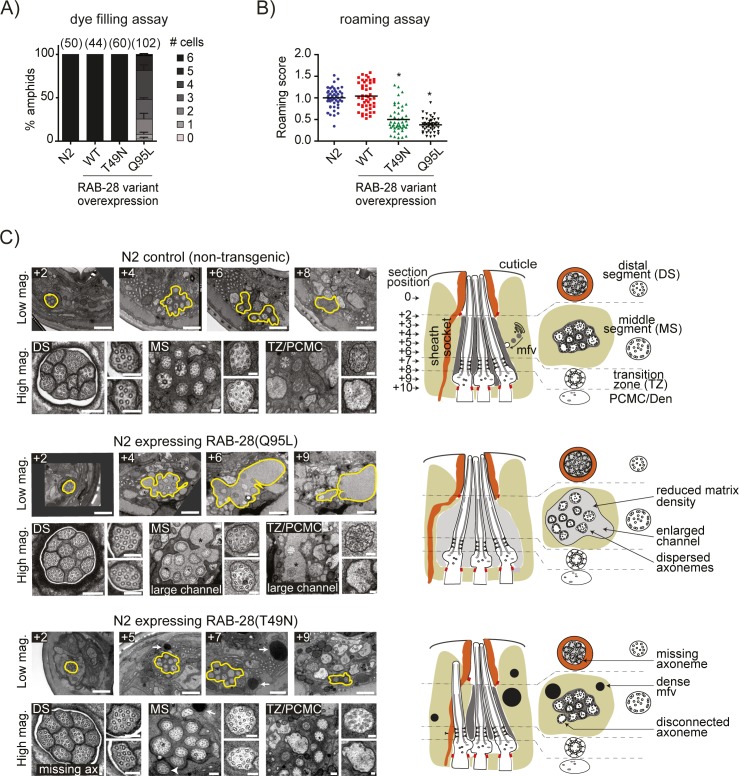
Worms overexpressing RAB-28(GTP) or RAB-28(GDP) display overlapping and distinct defects in sensory pore formation and function. **(A)** Dye filling (DiI) of amphid neurons in non-transgenic (control) or transgenic wild type (N2) worms expressing GFP::RAB-28(WT), GFP::RAB-28(GTP) or GFP::RAB-28(GDP). All constructs driven by the endogenous *rab-28* gene promoter and expressed to similar levels in transgenic animals (all injected at 5 ng/ul). For each amphid pore, the number of dye-filling neurons was scored. Each dataset represents mean ± standard deviation (error bars) from 3 independent experiments (at least 40 amphid pores scored for each strain per experiment). **(B)** Roaming scores for non-transgenic (control) and transgenic wild type (N2) worms expressing GFP-tagged RAB-28 variants. Scores normalised to non-transgenic N2 worms. For each strain, >45 worms were scored. *p<0.01 (unpaired Student t-test vs non-transgenic N2 controls). **(C)** Transmission electron microscopy images of the amphid pore from serial cross-section of wild type (N2) and N2 worms expressing GFP-tagged RAB-28(GTP) or RAB-28(GDP). Low magnification images (top rows) show the enlarged amphid channel (yellow outline and asterisk) in RAB-28(GTP)-expressing worms and dense matrix filled vesicles (mfv) in the amphid sheath cell of RAB-28(GDP) expressing worms (white arrows). High magnification images (bottom rows) display close ups of the amphid pore and ciliary axonemes, showing the distal segments (DS), middle segments (MS), transition zones (TZ) and periciliary membrane compartments (PCMC). Note the misplaced (disconnected) channel axoneme (white arrowhead) that fails to enter the channel of worms expressing RAB-28(GDP). Images representative of at least 4 analysed pores for each strain. Cartoons show the amphid channel in cross section and longitudinal orientations (only 3 of the 10 axonemes shown for simplicity in longitudinal cartoon), and indicate observed phenotypes. Numbers above images indicate the position of the section relative to the most anterior section (at ‘0’); section positions also indicated in cartoon. Scale bars; 1 μm (top rows); 200 nm (large images in bottom rows); 100 nm (small images in bottom rows).

The differences in localisation and IFT behaviour between the constitutively inactive (GDP-locked) and active (GTP-locked) forms of RAB-28 prompted us to investigate cilium structure and function in worms overexpressing these small GTPase variants. Assessment of dye-filling reveals reduced levels of dye uptake in the amphid neurons of wild type worms expressing RAB-28(GTP), compared to normal dye-filling for wild type worms expressing RAB-28(WT) or RAB-28(GDP) (Fig 5A). Similar dye-filling results were obtained when these variants were expressed in *rab-28(gk1040)* worms, although worms expressing RAB-28(GDP) may display a very weakly penetrant phenotypic defect (S8A Fig). Despite the distinct dye-filling phenotypes, worms expressing RAB-28(GTP) or RAB-28(GDP) both show a reduced roaming behaviour (Fig 5B), suggestive of cilia-related sensory abnormalities [78].

To investigate the structural basis of these defects, we examined amphid pore ultrastructure in wild type (N2) worms expressing GFP-tagged RAB-28(GTP) or RAB-28(GDP). Somewhat surprisingly, despite the dye uptake phenotype, RAB-28(GTP)-expressing amphid channel cilia are of normal length and morphology, with intact middle and distal segments, as well as TZ compartments (Fig 5C). However, an increased number of ciliary axonemes possess B-tubule seam breaks (‘unzipped’ microtubules), indicative of incomplete protofilament formation (S8B Fig). A more striking phenotype is that the proximal region of the sheath cell-defined amphid channel is greatly enlarged, and the ciliary axonemes are less tightly bundled (Fig 5C). Also, the channels of RAB-28(GTP) expressing worms display reduced electron densities, potentially indicating abnormally low concentrations of matrix material, which is thought to be secreted by the sheath cell (Fig 5C) [43]. In contrast, although the amphid channel in RAB-28(GDP)-expressing worms is not expanded, these worms exhibit a number of abnormally large dense matrix-filled vesicles (MFVs) in the sheath, in that portion of the cell surrounding the channel (Fig 5C). Such electron dense vesicles are not found in the channel region of the amphid sheath cell of wild type (N2), *rab-8(gk1040)* or RAB-28(GTP) overexpressing worms (Fig 5C), indicating this phenotype is specific to RAB-28(GDP) overexpression. A second phenotype unique to RAB-28(GDP)-expressing worms is that one channel axoneme is missing from the amphid pore, and instead lies adjacent to the channel, embedded in the sheath (Fig 5C).

Together, these findings indicate that overexpression of constitutively active or inactive RAB-28 cause variant-specific defects in the amphid sheath (enlarged channel; dense MFV accumulation) and ciliated cells (dye-filling defect; misplaced amphid channel axoneme), although shared phenotypes are also observed (roaming defect). The sheath cell phenotypes likely represent a cell non-autonomous function because our transcriptional reporter for RAB-28 (GFP under the control of the *rab-28* promoter) is not expressed in the sheath cell (S9 Fig). Thus, we conclude from these overexpression data that RAB-28 serves ciliated cell autonomous and sheath cell non-autonomous roles in sensory pore formation and function.

## Discussion

In this study, we identified a specific *C*. *elegans* ciliary gene expression profile using a set of RNA-Seq libraries, originally produced to annotate the transcriptome, that together provide a temporal landscape of whole-organism gene expression [45–47]. A major advantage of *C*. *elegans* as a model for ciliary biology is that ciliogenesis occurs during a temporally discrete window of embryonic development (Fig 1) [48], making it well suited for this type of temporal gene expression analysis. The expression profile for known cilia genes peaks at the onset of ciliogenesis and decreases post-ciliogenesis, likely to levels adequate to maintain cilium structure. Using this pattern, we uncover new ciliary associations for several conserved, but poorly characterised proteins, including the orthologues of YAP, MAP9 and CCDC149. The ciliary localisation of MAPH-9 (microtubule-associated protein 9) is consistent with a known functional association between MAP9 and the centrosomal regulatory kinase PLK1 [72], the centrosomal localisation and suggested ciliary functions for MAP9 in zebrafish [69], and a role for MAP9 as a modifier of retinal degeneration in Dachshunds [70]. YAP also has previous links to cilia, including a role in ciliogenesis [67,68] as well as being regulated by the ciliary gate protein, NPHP4 [71]. Although we do not understand the exact ciliary functions of MAP9 and YAP, their suggested roles in other contexts include regulation of ciliary microtubules (MAP9) and cilium-based signalling (YAP) [67–72]. Since 64 of the 185 genes in our predicted list of ciliary genes (cluster 1) possess known ciliary associations, we are confident that additional cilia-related components can be uncovered from this dataset.

**Fig 6:**
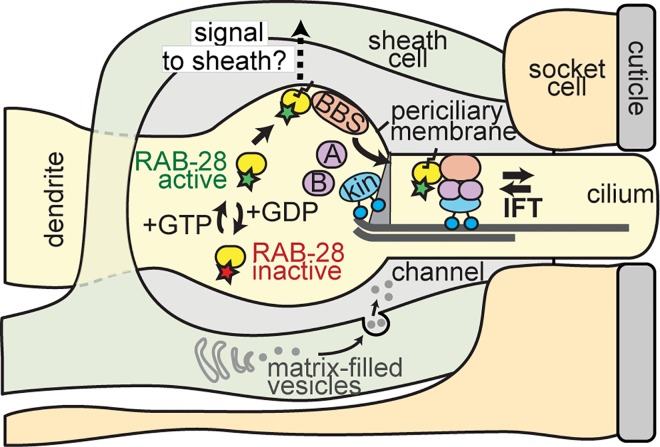
Model of RAB-28 ciliary associations in *C*. *elegans*. Shown is a cartoon of the amphid sensory pore. The ciliary axonemes (only one shown for simplicity) invaginate through the sheath cell process into a matrix-filled channel. The process of the socket cell forms a doughnut-like ending that establishes the distal part of the channel, by sitting on top of the sheath cell process. Binding of GTP to RAB-28 promotes its targeting to the periciliary membrane (PCM) via interactions with the ciliary base-associated BBSome complex. RAB-28(GTP)-BBSome assemblies associate with the IFT machinery at the ciliary base and enter the cilium via IFT-directed movement. Activated RAB-28 is proposed to serve cell non-autonomous functions by regulating the release (or cell surface expression) of a neuronal signal that controls sheath cell channel morphogenesis. In worms overexpressing RAB-28(GTP) or RAB-28(GDP), potentially opposing amphid channel size phenotypes are observed (enlarged vs small), together with modest cilium structure and sensory behaviour abnormalities (not shown in above model; see Fig 5 for details).

One of the highest ranked hits in our candidate list of ciliary genes is RAB-28, a small GTPase whose human counterpart is linked to autosomal-recessive cone-rod dystrophy and vision impairment [62,63]. Although recent studies show that mammalian RAB28 is localised to the mammalian photoreceptor basal body and cilium [62,64], its role in photoreceptors and in other tissues and organisms remains unknown. Here, we show that *C*. *elegans* RAB-28 is specifically expressed in ciliated sensory neurons, and undergoes IFT. RAB-28 IFT association depends on its GTPase activity; whereas the GDP-locked inactive form localises diffusely with little or no observable IFT, the GTP-locked active form concentrates almost exclusively at the periciliary membrane and undergoes IFT. Moreover, the periciliary membrane association and IFT behaviour of active RAB-28 depends on the BBSome, which functions as an IFT cargo-adaptor [11,14,85]. We conclude that *C*. *elegans* BBSome complexes recruit activated RAB-28 to the periciliary membrane, and regulate docking to IFT trains (Fig 6).

Our findings also demonstrate that RAB-28 is probably not a ‘core’ component of the BBSome or IFT particles, since ciliary structures, as well as BBSome and IFT-subcomplex A/B protein localisations, are unaffected in *rab-28* mutant worms. Instead, RAB-28 may associate with these complexes as part of a ‘peripheral’ submodule, performing auxiliary functions unrelated to ciliogenesis, such as those described for metazoan IFT27 (Rab-like 4; RABL4), ARL6 (BBS3) and RAB8, whose ciliary targeting or removal also depend on GTP binding and the BBSome (ARL6, RAB8) [86–89]. Consistent with a non-essential global requirement for ciliogenesis, IFT and BBSome integrity, patients carrying predicted strong loss-of-function or possibly null alleles in *RAB28* do not present with wider ciliopathy symptoms beyond cone-rod dystrophy [62,63].

### RAB-28 cell non-autonomous function

Another revealing observation from our work is that overexpression of constitutively active (GTP-locked) or inactive (GDP-locked) RAB-28 cause variant-specific defects in the non-ciliated amphid sheath cell that supports the ciliated endings of amphid neurons. Specifically, RAB-28(GTP) overexpression causes an enlargement of the sheath cell-defined portion of the amphid sensory pore, whereas RAB-28(GDP) overexpression causes the accumulation of abnormally dense matrix-filled vesicles (MFVs) in the sheath cell. The RAB-28 variant constructs, as well as the RAB-28(WT) construct, are expressed at similar levels, using the same promoter sequence; thus, the observed variant-specific phenotypes are not likely due to differences in expression level, but instead are linked to the nucleotide bound state of RAB-28. Most interestingly, the abnormal sheath cell phenotypes appear to reflect a cell-non autonomous effect because RAB-28 is expressed in ciliated sensory neurons, and not in the amphid sheath cell (**S9 Fig**).

In *C*. *elegans*, the amphid channel is fashioned from glial cell (sheath and socket) processes which extend to the nose tip in close proximity to the dendritic processes of the ciliated sensory neurons. The proximal part of the channel forms from a hole in the sheath process which the channel cilia fully penetrate, whereas the distal part of the channel derives from a doughnut-shaped ending of the socket process that fuses with the cuticle and the underlying sheath (**Fig 5C**). The formation and function of neurons and glia in the amphid pore appear tightly linked, with channel morphogenesis thought to depend on signals from both cell types [43,44,90]. It is also suggested that amphid channel size is regulated via a balance of ‘exocytic’ membrane delivery via MFVs (increases channel size) and ‘endocytic’ membrane retrieval (reduces channel size) at the sheath cell plasma membrane [91]. By integrating this model with our findings, we hypothesise that constitutively active RAB-28 may enhance MFV delivery to the sheath cell membrane, leading to an enlarged channel, whereas constitutively inactive RAB-28 blocks MFV delivery and results in abnormally dense MFVs in the sheath. One prediction of this model is that expression of constitutively inactive RAB-28 would reduce amphid channel size, and consistent with this idea, one channel axoneme fails to enter the amphid pore of RAB-28(GDP) expressing worms.

How RAB-28 expressed in ciliated amphid neurons might control morphogenesis events in the supporting non-ciliated sheath cell is unclear. One scenario is that RAB-28 regulates extracellular release of neuronal factors purported to signal to the sheath cell during channel morphogenesis in the embryo [42–44] (Fig 6). In support of this notion, activated RAB-28 is almost exclusively concentrated at the periciliary membrane, which is the site of ectosome release in certain nematode sensory neurons; also, RAB-28 expression is elevated in nematode ectosome-releasing cells [92,93]. Future work will be required to investigate possible roles for RAB-28 in cilia-related ectosome release, and to identify sheath cell morphogenic factors released by the ciliated neurons.

A surprising observation is that sensory pore structure and function appears grossly normal in the *rab-28* deletion mutant, which is likely a null allele. This indicates that other genes or pathways can compensate for *rab-28* loss-of-function, but that the phenotypic effects of the overexpressed dominant active and inactive RAB-28 variants are more severe and cannot be as easily compensated. One possibility is that the cilium structure, and cilium-dependent behavioural phenotypes in worms overexpressing RAB-28(GTP) and/or RAB-28(GDP) derive from excessive activation of downstream effectors or possibly aberrant sequestration of GTPase activating proteins (GAPs) or GDP-GTP exchange proteins (GEFs) common to RAB-28 and other ciliary GTPases. To fully decipher the mechanistic basis of the RAB-28 gain vs loss of function phenotypes, and the physiological relevance of our findings, future efforts should include an examination of worms expressing RAB-28 variants expressed at endogenous or near-endogenous levels and the identification of currently unknown regulators of RAB-28 (GEF and GAP proteins).

### Possible functional relationship between RAB28 and IFT27?

Given the close sequence similarity between RAB28 and IFT27, and some of their common functional properties discussed below, we suggest that these two RAB GTPases may have similar or partially overlapping ciliary functions. RAB28 is conserved across metazoans and many ciliated protists (S10 Fig), and inferred to be one of 23 founding RAB family members present in the last eukaryotic common ancestor (LECA) [94,95]. Phylogenetically, RAB28 is most closely related to IFT27 (RABL4), which likely also existed in LECA and is dispersed throughout the eukaryotic lineage [94,95] (S10 Fig). Like the majority of ciliary and IFT components, both RAB28 and IFT27 are restricted to organisms that have cilia (S10 Fig). Interestingly, although RAB28 and IFT27 are present in most metazoans and many protists (*e*.*g*., *Chlamydomonas* and *Trypanosoma* have both), neither protein appears to be present in *Drosophila* and IFT27 is missing from *C*. *elegans* (S10 Fig).

RAB28 and IFT27 appear to share overlapping functional properties. As we show for nematode RAB-28, Trypanosome and mammalian IFT27 associate with IFT in a GTP-dependent manner [87,88,96]. Also, cilium formation is unaffected in mammalian cells with null mutations in IFT27 (similar to *C*. *elegans rab-28* mutants), although disruption of IFT27 in protists results in short flagella [74,96]. In addition, mutations in IFT27 cause BBS, displaying a broad spectrum of ciliopathy phenotypes, including the cardinal retinal degeneration phenotype observed in RAB28 patients [97,98]. Furthermore, both small GTPases are functionally associated with the BBSome, albeit in different ways. The localisation and IFT motility of nematode RAB-28 is BBSome-dependent, but not *vice versa* (*i*.*e*., BBS-5 localisation is normal in *rab-28* mutants), whereas mammalian IFT27 regulates BBSome assembly and removal from the cilium. When taken together, the above observations could suggest that in vertebrates RAB28 and IFT27 function at distinct steps of a common pathway, potentially ‘upstream’ (IFT27) and ‘downstream’ (RAB-28) of the BBSome [87,88]. Since IFT27 and the BBSome remove proteins from cilia [85,87,88,99], RAB28 may perform a similar function by facilitating the BBSome-mediated removal of a more-restricted set of cargo.

In further support of a common, yet tissue-specific, transport role for related ciliary GTPases such as IFT27 and RAB28, it is noteworthy that disruption of the short ARL6/BBS3 isoform in mice and zebrafish results in broad spectrum BBS-related phenotypes, whereas disruption of the long isoform causes a retina-specific phenotype [100]. Transition zone-localised RPGRIP1 and RPGRIP1L represent additional examples of very closely related ciliopathy proteins linked to a broad tissue pathogenesis (RPGRIP1L), versus a retinal-specific disease (RPGRIP1) [101–104]. Finally, whereas IFT27 is known to regulate the ciliary localisation of sonic hedgehog proteins [87,88], the identity of RAB28-associated cargoes is currently unknown. Interestingly, RAB28 itself may be a cargo of the prenyl-binding protein PDE6D (cGMP phosphodiesterase delta subunit) which immunoprecipitates RAB28 [105]; in agreement with this possibility, loss of PDE6D in the mouse phenocopies the photoreceptor degeneration of RAB28 patients [106]. In light of our worm data, it is possible that PDE6D delta functions with the BBSome, either together or at distinct parts of a transport pathway, to target RAB28 to ciliary membranes.

### Concluding remarks

Our study describes a novel method for identifying genes with ciliary functions that complements other studies (bioinformatics, genomics, proteomics) aimed at deciphering a complete ‘ciliome’. From our list of candidates, we uncover several novel, evolutionarily-conserved cilium-associated proteins. Our in-depth analysis of RAB-28 in *C*. *elegans* reveals it to be a novel IFT- and BBSome-associated protein with cell autonomous and non-autonomous roles in maintaining the structural and functional integrity of cilia, and of the sensory neuron-supporting cells. Further analyses of mammalian RAB28 are required to reveal its functional similarities and differences with IFT27, and possible functional associations with PDE6D and ARL6, to help explain its seemingly specific function in ciliary photoreceptors, and thus specific association with cone-rod dystrophy.

## Materials and Methods

### Identifying genes with expression correlated with known ciliary genes

We curated a list of 41 *C*. *elegans* genes with established roles in ciliary function and used these to search for genes with correlated expression. These “bait” genes included: R148.1, F38G1.1, Y75B8A.12, F20D12.3, Y41G9A.1, R01H10.6, C54G7.4, C27A7.4, R31.3, C48B6.8, F32A6.2, C27H5.7, T28F3.6, Y37E3.5, F59C6.7, F19H8.3, ZC84.2, F35D2.4, H01G02.2, B0240.3, ZK520.3, C02H7.1, ZK418.3, K08D12.2, F23B2.4, Y105E8A.5, F46F6.4, K07G5.3, F33H1.1, F02D8.3, K03E6.4, C38D4.8, T26C12.4, T27B1.1, Y110A7A.20, F53A9.4, M04C9.5, C30B5.9, C09G5.8, R13H4.1 and F54C1.5. We used expression data derived from RNA-seq for seven developmental stages (EE = early embryo, LE = late embryo, L1 = mid-L1 larvae, L2 = mid-L2 larvae, L3 = mid-L3 larvae, L4 = mid-L4 larvae and YA = young adult) obtained from [45–47]. As the expression values were relative to individual isoforms, we first computed an average RPKM (reads per kilobase per million) value for each gene across all isoforms. Using these 41 genes, we next computed the pairwise Pearson correlation of each gene against all 20,363 *C*. *elegans* genes in the data set across all libraries and estimated a P value for each test. As we were interested in capturing genes with expression correlates to any of these known ciliary genes, we retained the smallest P value from these 41 tests for subsequent calculations. To avoid circularity, we retained the second-smallest P value for correlation tests involving one of the bait genes. In addition to direct correlation, we sought to identify genes exhibiting a developmental expression pattern similar to known ciliary genes. We observed that all ciliary genes exhibit at least a 10-fold reduction in expression level (relative to their peak expression level) in the latter three developmental stages. A total of 5960 genes showed this expression trend.

Following the identification of those genes exhibiting strongly correlated expression to any of the known ciliary genes, this set was further clustered to search for sub-groupings of genes with strongly coordinated expression. The distance between individual genes was calculated using the ‘dist’ function in R and hierarchical clustering was performed using single, average and complete linkage. S1 Fig shows a heat map representation of the expression for each of the candidate ciliary genes with the dendrogram derived from clustering using complete linkage and cluster 1 at the top.

### Chemosensory expression pattern and promoter element prediction

Using the method described above, we used *srg-36* as a bait to identify co-expressed genes. We found 80 genes (p < 0.001), including 27 other predicted chemoreceptors [52]. The 1000 bp upstream from the start codon of all 28 chemoreceptors (including the bait *srg-36*) were entered into the program MEME [107,108], which found a consensus that matches the published E-box sequence [54]. Separately, the 1000 bp upstream of all the conserved cluster 1 were entered into the MEME suite program DREME [107,108], which found several potential transcription factor binding sites. We used the MEME suite program TomTom to compare identified sites to known sites [108]. Identified promoter elements were compared to consensus splice and trans-splice sites and any that matched were subsequently removed.

### *C*. *elegans* maintenance and crossing

All nematode strains were maintained and cultured at 20°C on nematode growth medium (NGM) plates seeded with OP50 *Escherichia coli* using standard techniques. Standard genetic crossing techniques were used to introduce transgenes into genetic backgrounds. The *rab-28(gk1040) mksr-1(ok2092)*, *mksr-2(tm2452)*, *mks-5(tm3100)*, *mks-3(tm2547)*, *mks-6(gk674)*, and *nphp-4(tm925)* mutations were followed using genotyping PCR (primer sequences available upon request).

### Strains

N2 (Bristol)

rab-28(gk1040)

che-11(e1810)

bbs-8(nx77)

*N2;oqEx300[*P*rab-28*::*gfp +* P*unc-122*::*gfp]*

*N2;oqEx301[*P*rab-28*::*gfp*::*rab-28 +* P*unc-122*::*gfp]*

*N2;oqEx302[*P*rab-28*::*gfp*::*rab-28(T49N) +* P*unc-122*::*gfp]*

*N2;oqEx303[*P*rab-28*::*gfp*::*rab-28(Q95L) +* P*unc-122*::*gfp]*

*N2;oqEx304[*P*rab-28*::*gfp*::*rab-28(Q95L) +* P*unc-122*::*gfp]*

*bbs-8(nx77);oqEx301[*P*rab-28*::*gfp*::*rab-28 +* P*unc-122*::*gfp]*

*bbs-8(nx77);oqEx302[*P*rab-28*::*gfp*::*rab-28(T49N) +* P*unc-122*::*gfp]*

*bbs-8(nx77);oqEx303[*P*rab-28*::*gfp*::*rab-28(Q95L) +* P*unc-122*::*gfp]*

*bbs-8(nx77);oqEx304[*P*rab-28*::*gfp*::*rab-28(Q95L) +* P*unc-122*::*gfp]*

*che-11(e1810);oqEx301[*P*rab-28*::*gfp*::*rab-28 +* P*unc-122*::*gfp]*

rab-28(gk1040);oqEx58[arl-13::gfp + rol-6(su1006)]

rab-28(gk1040);Is[osm-6::gfp]

rab-28(gk1040);myIs[pkd-2::gfp]

rab-28(gk1040);nxEx289[rpi-2::gfp + xbx-1::tdTomato + rol-6(su1006)]

rab-28(gk1040);nxEx475[bbs-5::gfp + pCeh361]

N2;nxEx869[fam-161::gfp + xbx-1::tdTomato + rol-6(su1006)]

*N2;nxEx1157[*P*bbs-8*::*gfp*::*rab-28 + xbx-1*::*tdTomato + rol-6(su1006)]*

N2;nxEx250[rpi-1::gfp + xbx-1::tdTomato + rol-6(su1006)]

N2;nxEx1223[maph-9::gfp + xbx-1::tdTomato + rol-6(su1006)]

*N2;nxEx1230[*P*bbs-8*::*gfp*::*yap-1 + xbx-1*::*tdTomato + rol-6(su1006)]*

*N2;nxEx2619[*P*ccdc-149*::*gfp*::*ccdc-149 + xbx-1*::*tdTomato + rol-6(su1006)]*

*N2;nxEx2623[*P*ccdc-104*::*gfp*::*ccdc-104cDNA + xbx-1*::*tdTomato + rol-6(su1006)]*

*N2;nxEx2626[*P*ccdc-149*::*gfp + xbx-1*::*tdTomato + rol-6(su1006)]*

*N2;nxEx2628[*P*ccdc-104*::*gfp + xbx-1*::*tdTomato + rol-6(su1006)]*

N2;nxEx654[tza-3::gfp + xbx-1::tdTomato + rol-6(su1006)]

mks-6(gk674);nxEx654[tza-3::gfp + xbx-1::tdTomato + rol-6(su1006)]

nphp-4(tm925);nxEx654[tza-3::gfp + xbx-1::tdTomato + rol-6(su1006)]

mks-3(tm2547);nxEx654[tza-3::gfp + xbx-1::tdTomato + rol-6(su1006)]

mks-5(tm3100);nxEx654[tza-3::gfp + xbx-1::tdTomato + rol-6(su1006)]

mksr-1(ok2092);nxEx654[tza-3::gfp + xbx-1::tdTomato + rol-6(su1006)]

mksr-2(tm2452);nxEx654[tza-3::gfp + xbx-1::tdTomato + rol-6(su1006)]

### Dye-filling and sensory behavioural assays

For the dye-filling assay [109], worms were incubated in 200 μl of DiI solution (Invitrogen; diluted 1:200 with M9 buffer) for 30 min. After incubation, worms were recovered on seeded nematode growth medium plates for a further 30 min and then mounted on slides. Epifluorescence wide-field imaging under the red filter was used to image DiI uptake into the ciliated amphid and phasmid cells. For the roaming (foraging) assay, single worms were placed for 18 hours onto seeded NGM plates and track coverage assessed using a grid reference [109]. For the osmotic avoidance assay, 5–6 worms were placed within a ring of 8M glycerol (Sigma) supplemented with Bromophenol Blue (Alfa Aesar) on unseeded NGM plates and observed for 10 min. Worms that crossed the barrier were removed from the assay. Chemotaxis attraction assays (towards isoamyl alcohol) were performed as previously reported [109], with a chemotaxis index calculated at 30 min and 60 min.

### Fluorescent reporter construction and transgenic strain generation

All constructs were generated by PCR fusion [110]. For the transcriptional (promoter) P*rab-28*::*gfp* construct, *gfp* amplified from pPD95.67 was fused to an 432-bp fragment of the 5’UTR of *rab-28* that included the first 14 bp of exon 1 (start codon thymine mutated to guanine). For the transcriptional reporters for P*ccdc-104* and P*ccdc-149*, *gfp* amplified from pPD95.77 was fused to 1997 and 395 bp, respectively, of the 5’UTR for each gene. For the translational (promoter + protein) *fam-161*, *rpi-1*, *maph-9*, *ccdc-149* and *tza-3* reporters, the entire genomic sequence (exons and introns) were fused to *gfp* including promoter sequences of 1373 bp, 2317 bp, 973 bp, 395 bp and 457 bp, respectively. For the translational reporter for *rab-28* and *yap-1* in Fig 2, 341 bp of the *bbs-8* promoter followed by *gfp* were fused to the genomic sequence including 3’ UTR for each gene. For the *ccdc-104* translational reporter, 1997 bp of the *bbs-8* promoter followed by *gfp* were fused to the *ccdc-104* cDNA and 3’ UTR. For the translational P*rab-28*::*gfp*::*rab-28* reporter (Figs 3 and 4), the entire genomic sequence and 970-bp of the 3’UTR of *rab-28* was first fused 5’ to a *gfp* fragment, amplified from pPD95.77. The resulting *gfp*::*rab-28* amplicon was subsequently fused to a 422-bp fragment consisting of the 5’UTR (promoter) sequence of *rab-28*. To make the P*rab-28*::*gfp*::*rab-28*(GDP) and P*rab-28*::*gfp*::*rab-28*(GTP) constructs, primers incorporating the corresponding T49N and Q95L mutations were first used to amplify 5’ and 3’ fragments of the *rab-28* genomic sequence, and these were fused by PCR to establish the *rab-28*(T49N) and *rab-28*(Q95L) amplicons. These amplicons were subsequently fused to a P*rab-28*::*gfp* fragment, generated by fusing the *rab-28* promoter sequence (see above) to *gfp* amplified from pPD95.77. Transgenic worms expressing the above constructs were generated by gonadal transformation of N2 hermaphrodites via microinjection and subsequent screening for transgenic progeny. *rab-28* constructs were injected at a concentration of either 5 ng/μl (all translational constructs except *oqEx304* which was injected at 0.5 ng/μl) or 50 ng/μl (transcriptional constructs), together with a coelomocyte (P*unc-122*::*gfp*) or pRF4 (*rol-6(su1006)*) co-injection marker at 50–100 ng/μl.

### *C*. *elegans* fluorescence microscopy, IFT motility and FRAP assays

Strains in Fig 2 and S4B Fig were anaesthetised with 10 mM levamisole in M9 buffer, mounted on slides with 8% agarose pads, and observed by epifluorescence or spinning-disc (WaveFX; Quorum Technologies) confocal microscopy performed on an Zeiss Axio Observer Z1 with a Hamamatsu 9100 EMCCD camera. Image capture and visualisation were performed on Volocity (PerkinElmer). All other worms were immobilised with 40mM tetramisole (Sigma no. L9756) or microbeads (Polysciences no. 00876–15) and mounted on 4% or 10% agarose pads. Epifluorescence images were taken on an upright Leica DM5000B and confocal images on an inverted Nikon Eclipse Ti microscope with a Yokogawa spinning-disc unit (Andor Revolution). Images were acquired using a charge-coupled device camera (iXon+EM-CCD, Andor Technology) and analysed using Image J software. For IFT assays, time-lapse (multi tiff) movies of IFT along phasmid cilia were taken at 200 ms exposure and 4 fps. Separated anterograde and retrograde kymographs were generated from multi tiff files using Icy image analysis software (http://icy.bioimageanalysis.org/) and rates determined using ImageJ [111]. Fluorescence recovery after photobleaching (FRAP) assays were performed using the above confocal microscope with an attached FRAPPA unit (Andor Technology). Samples were bleached using a single pulse of the 488nm laser at 100% with a dwell time of 100 μs. Images were recorded immediately post-bleach and continuously thereafter. Fluorescence intensities measured with image J software following a recently described protocol [109]. Values were normalised to pre-bleach values and corrected for signal intensity loss during image acquisition.

### Transmission Electron Microscopy

Young adult worms were fixed, sectioned and imaged as previously reported [109].

## Supporting Information

S1 Fig Clustering of the bait and target genes with the cilia-related expression pattern. Genes with a cut-off of p<1e-4 were selected for further clustering based on their expression profiles. Most of the 41 baits (signified by the blue horizontal lines on the left side of the heat map) used to identify potential cilia genes cluster together at the top for a total of 185 candidate ciliary genes. EE; early embryo. LE; late embryo. L1; larval stage 1. L2; larval stage 2. L3; larval stage 3. L4; larval stage 4. YA; young adult.
(TIF)

S2 Fig Promoter elements identified in the conserved cluster 1 genes. Schematics of promoter elements in the temporally co-expressed, conserved cluster 1 genes, as modified from the MEME output [108]. Gene name to left of motifs indicate a significant match to a previously identified promoter element.
(TIF)

S3 Fig E-box promoter elements in serpentine chemoreceptors. **(A)** E-box schematic for the serpentine chemoreceptors as modified from the MEME output [107,108].
**(B)** List of E-box elements in the promoters of the serpentine chemoreceptors as modified from the MEME output [107,108].
(TIF)

S4 Fig Enriched or exclusive expression of *rab-28*, *ccdc-104 and ccdc-149* transcriptional GFP reporters in ciliated cells, and association of TZA-3 with the MKS module at the transition zone. **(A)** Representative head and tail images of worms expressing transcriptional GFP reporters under the control of the indicated gene promoter (P). Worms expressing P*rab-28*::GFP were co-stained with DiI to label 6 pairs of amphid (head) ciliated neurons and the pair of phasmid (tail) ciliated neurons. Worms with P*ccdc-104*::GFP or P*ccdc-149*::GFP were co-expressed with XBX-1::tdTomato to identify ciliated cells. Scale bars; 15 μm. **(B)** Localisation of a translational TZA-3::GFP reporter with the ciliary protein XBX-1::tdTomato. TZA-3 localises to the transition zone (TZ) in N2 wild type (wt) amphids and phasmids. TZA-3 remains at the TZ in *mks-3*, *mks-6* and *nphp-4* mutants. However, strong localisation to the TZ is not observed in *mksr-1* and *mksr-2* mutants, and loss of TZA-3 ciliary base localisation is seen in the *mks-5* mutant. Scale bar; 5 μm.
(TIF)

S5 Fig Cilium structure, function and transport in the *rab-28 (gk1040)* mutant. **(A)** Schematic of the *rab-28* gene model and location of the *gk1040* deletion. Exons denoted by boxes. Numbering refers to genomic nucleotide positions from the start codon in exon 1. Deletion breakpoints (781–1776) determined via Sanger sequencing. *gk1040* removes the critical GTP-binding switch II domain and the CAAX box essential for RAB protein membrane association.
**(B)** Representative images of the head and tail regions of N2 wild type and *rab-28(gk1040)* mutant worms following a DiI incorporation assay into amphid and phasmid neurons. Scale bar; 20 μm.
**(C)** Representative images of amphid and phasmid cilia from N2 wild type and *rab-28(gk1040)* worms expressing OSM-6::GFP. Scale bars; 2 μm.
**(D)** Transmission electron microscopy images of the amphid pore from serial cross sections of N2 wild type and *rab-28(gk1040)* worms. Low (large panels) and high (small panels) magnification images are shown. Images representative of at least 4 analysed pores for each strain. Both worms show 10 ciliary axonemes in the amphid pore, with each axoneme consisting of a distal segment (DS), middle segment (MS), transition zone (TZ) and periciliary membrane compartment (PCMC). Cartoon shows the amphid channel in cross section and longitudinal orientations (only 3 of the 10 axonemes shown for simplicity in longitudinal cartoon). Numbers above images indicate the position of the section relative to the most anterior section (at ‘0’); section positions also indicated in cartoon. Scale bars; 200 nm (large panels); 100 nm (small panels).
**(E)** Assessment of *rab-28(gk1040)* cilia-related sensory behaviours. Shown is a population-based isoamyl alcohol (IAA) attraction assay (n = 8 for N2 wild type and *osm-5*; n = 12 for *rab-28*), as well as single worm assays that measure roaming (n = 35 for all strains) and osmotic avoidance (n = 86 for N2; n = 37 for *osm-5*; n = 70 for *rab-28*) behaviours. % avoidance (fraction of worms that avoid -do not cross—the osmotic barrier) is plotted over the time course of the experiment (600 seconds). Chemoattraction index determined at two time points (30 & 60 minutes). *osm-5(p813)* worms used as a negative control. *p<0.01 (unpaired t-test vs WT control at the appropriate assay time point). Although *rab-28* worms present with a slightly reduced osmotic avoidance, this behaviour was not statistically significant compared with WT controls (log-rank and Mantel-Cox survival curve tests).
**(F)** *rab-28 (gk1040)* mutants are indistinguishable from wild-type worms with regards to the cilia-dependent phenotypes of carbon dioxide avoidance and body size. N2 wild-type and *rab-28(gk1040)* mutant worms respond statistically indistinguishably to a 4 sec puff of 10% CO_2_ delivered at 100s (represented by grey bar) by performing a burst of high-amplitude turns (repeated measures ANOVA, pNS). Error bars represent 95% confidence intervals. N2 and *rab-28 (gk1040)* mutant worms are statistically indistinguishable with regards to length along the midline of the body (ANOVA, pNS). For both graphs n = 112 worms (divided across 3 plates) for wild-type and n = 182 worms (divided across 3 plates) for the *rab-28 (gk1040)* mutant.
**(G)** Localisation of ciliary protein markers in *rab-28(gk1040)* worms. Representative images showing the localisation of ARL-13::GFP at the ciliary middle segments (MS) of phasmid (PHA/B) neurons, RPI-2::GFP at the periciliary membrane compartment (PCMC) of PHA/B neurons, PKD-2::GFP at the distal dendritic (DD) endings (including the cilium; cil) of male head (CEM) and tail (ray) neurons, and BBS-5::GFP at the ciliary base (BB) and along the axoneme (arrows indicate BBS-5 associated with moving IFT trains. BB; basal body, TZ; ciliary transition zone, DS; distal segment. Scale bars; 2 μm (ARL-13::GFP, RPI-2::GFP images) and 10 μm (PKD-2::GFP images).
(TIF)

S6 Fig Localisation of GFP-tagged RAB-28 variants in *rab-28*(*gk1040)* mutant. Representative images of entire phasmid tail neurons and the ciliary region of amphid head neurons from *rab-28(gk1040)* worms expressing GFP-tagged RAB-28(WT), RAB-28(GDP) or RAB-28(GTP). Like in a wild type background (Fig 3), all three markers are found in the cilium (cil), with RAB-28(GTP) highly enriched at the periciliary membrane. PCMC; periciliary membrane compartment (p). Den; dendrite Scale bars; 3 μm.
(TIF)

S7 Fig A GFP::RAB-28(Q95L) marker expressed at low levels localises exclusively to the periciliary membrane in a BBSome-dependent manner. Representative images of whole phasmid neurons and the ciliary region of amphid neurons from worms with an GFP::RAB-28(GTP) transgene (*oqEx304*) expressed at low levels. In the *rab-28(gk1040)* background, RAB-28(GTP) localises exclusively to the periciliary membrane, with weaker signals in the ciliary axoneme. In *bbs-8(nx77)* worms, no periciliary membrane enrichment is observed for RAB-28(GTP). Note that the *bbs-8* image has been overexposed to show the diffuse (non-specific) localisation of the faint RAB-28(GTP) signals. PCMC; periciliary membrane compartment. Den; dendrite. Scale bars; 3 um.
(TIF)

S8 Fig Dye-filling and B-tubule seam break phenotypes in wild type and *rab-28(gk1040)* worms overexpressing RAB-28(Q95L). **(A)** Dye filling of *rab-28(gk1040)* worms expressing the indicated GFP-tagged RAB-28 variant (WT, GDP-locked, GTP-locked). Non-transgenic N2 worms shown as a control for the dye-filling assay. For each amphid pore, the number of dye-filling neurons was scored. Each dataset represents mean ± standard deviation (error bars) from 3 independent experiments. At least 10 (N2) or 21 (all three RAB-28 variants) amphid pores scored for each strain per experiment. **(B)** Transmission electron microscopy images of the amphid pore from serial cross-sections showing an increased number of B-tubule seam breaks in the middle segments of age-matched (day 1 adult) wild type (N2) and N2 worms expressing GFP-tagged RAB-28(GTP). Shown are cross sections of the distal portions of the middle segment (section positions indicated in cartoon), where the B-tubule seam break phenotype occurs. For some of the sections, out of focus ultrastructure was obtained by tilting the sections as indicated. In wild type worms, B-tubule seam breaks are observed in 2 or possibly 3 axonemes (ADL, ASI [112]), whereas in worms expressing RAB-28(GTP), B-tubule seam breaks occur in at least 4–5 axonemes. Arrows indicate axonemes with clearly identifiable b-tubule seam breaks. Cartoon shows the amphid channel in longitudinal orientation (only 3 of the 10 axonemes shown for simplicity), and indicate observed phenotypes for the distal region of the middle segment. Scale bars; 200 nm.
(TIF)

S9 Fig RAB-28 is not expressed in the amphid sheath cell. Representative images from the amphid cell body region of worms co-expressing P*rab-28*::*gfp* (GFP under the control of the *rab-28* promoter; see also S4A Fig) and F16F9.3::dsRed, which is expressed exclusively in the amphid sheath cell [42]. Images show that GFP expression is restricted to ciliated neurons and does not include the sheath cell (outlined by white dotted line). This indicates that RAB-28 is not expressed in the amphid sheath cell. Scale bar; 15μm.
(TIF)

S10 Fig Phylogenetic distribution of RAB28 and RABL4 (IFT27) across eukaryotes. RAB28 and IFT27 protein orthologues are only found in eukaryotes that are ciliated during at least one life stage (ciliated cells are shown in green, non-ciliated cells in white). Green and white squares, respectively, denote the presence or absence of RAB28 or IFT27 in a given representative species (as presented in Elias *et al*. [95]).
(TIF)

S1 Table Sheet 1: List of all bait genes used to identify ciliary candidates. Sheet 2: List of all genes with given P and R values. Bait genes are highlighted yellow. Sheet 3: List of genes within filtered cluster 1 from the clustering analysis of cilia-related expression patterns. Sheet 4: Quantification of enrichment of cilia-related genes with the cilia-related expression pattern. Sheet 5: List of target and bait genes with the chemosensory-related expression pattern.
(XLSX)

S1 Movie Shown are 6 time-lapse videos of the phasmid cilia of N2 wild type, *che-11(e1810)* and *bbs-8(nx77)* worms expressing the indicated GFP::RAB-28 reporter (all under the control of the endogenous *rab-28* gene promoter). For illustration purposes, all videos played at 1.75x normal speed (original videos taken at 4 frames per second and played here at 7 frames per second). BB; basal body, cil; ciliary axonemes. Scale bars; 2 μm.
(MP4)

S2 Movie Time-lapse video from phasmid cilia of N2 wild type worms expressing GFP::RAB-28(GTP) at a low level (transgene *oqEx304*). RAB-28(GTP) localises almost exclusively near or at the periciliary membrane, with additional signals in the ciliary axoneme that move in both directions in an IFT-like manner. To make clear the IFT-like movement of RAB-28(Q95L) particles, the image was overexposed and the video played at 5x normal speed. Scale bar; 2 μm.
(MP4)
