# Supplementary figures and images for: Whole-Organism Developmental Expression Profiling Identifies RAB-28 as a Novel Ciliary GTPase Associated with the BBSome and Intraflagellar Transport
Source: PLoS Genet. 2016 Dec 8;12(12):e1006469. doi: 10.1371/journal.pgen.1006469 (PMC5145144; doi:10.1371/journal.pgen.1006469)

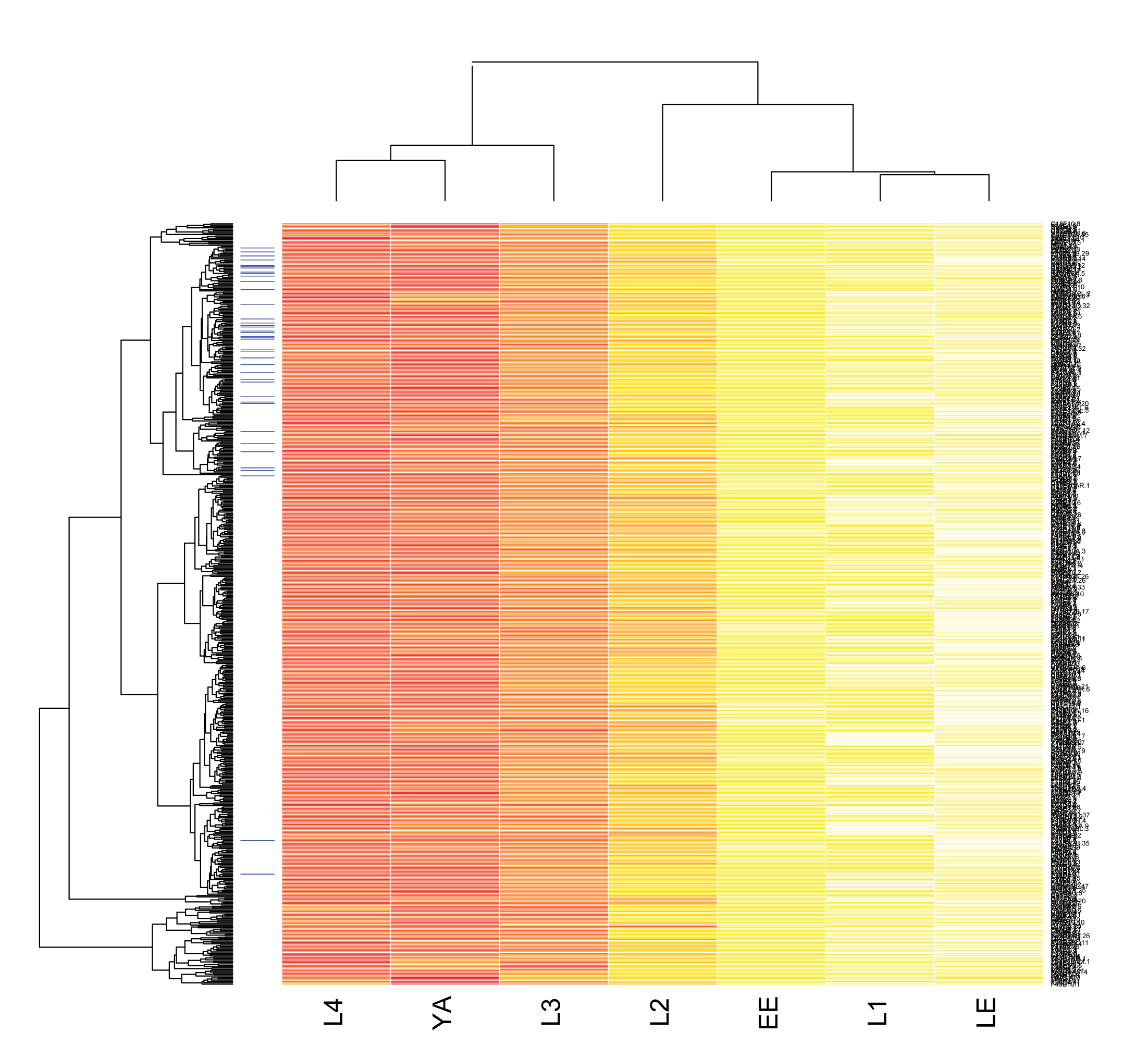

Supplement: S1 Fig — Genes with a cut-off of p<1e-4 were selected for further clustering based on their expression profiles. Most of the 41 baits (signified by the blue horizontal lines on the left side of the heat map) used to identify potential cilia genes cluster together at the top for a total of 185 candidate ciliary genes. EE; early embryo. LE; late embryo. L1; larval stage 1. L2; larval stage 2. L3; larval stage 3. L4; larval stage 4. YA; young adult. (TIF) [file pgen.1006469.s001.tif]

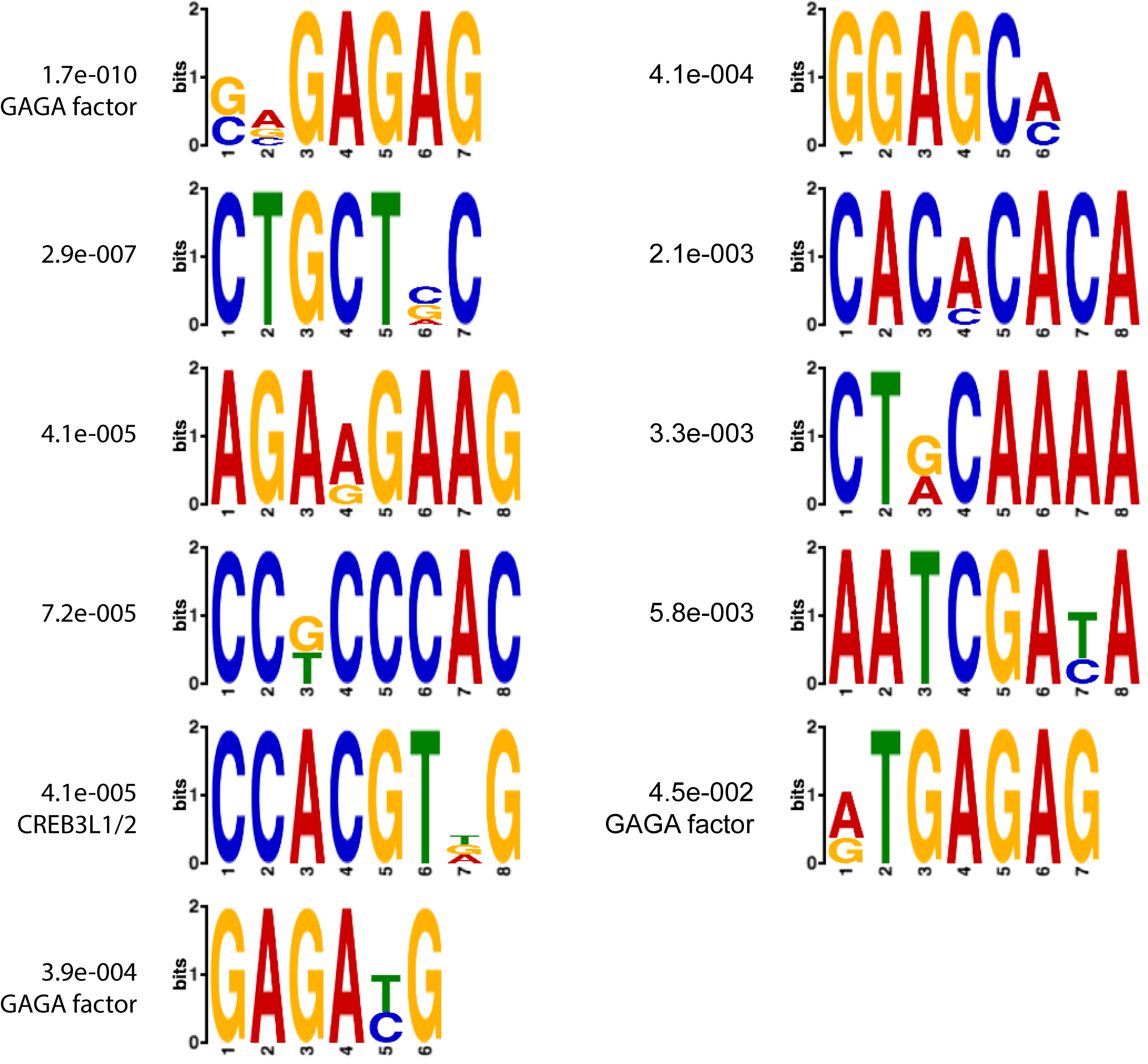

Supplement: S2 Fig — Schematics of promoter elements in the temporally co-expressed, conserved cluster 1 genes, as modified from the MEME output [108]. Gene name to left of motifs indicate a significant match to a previously identified promoter element. (TIF) [file pgen.1006469.s002.tif]

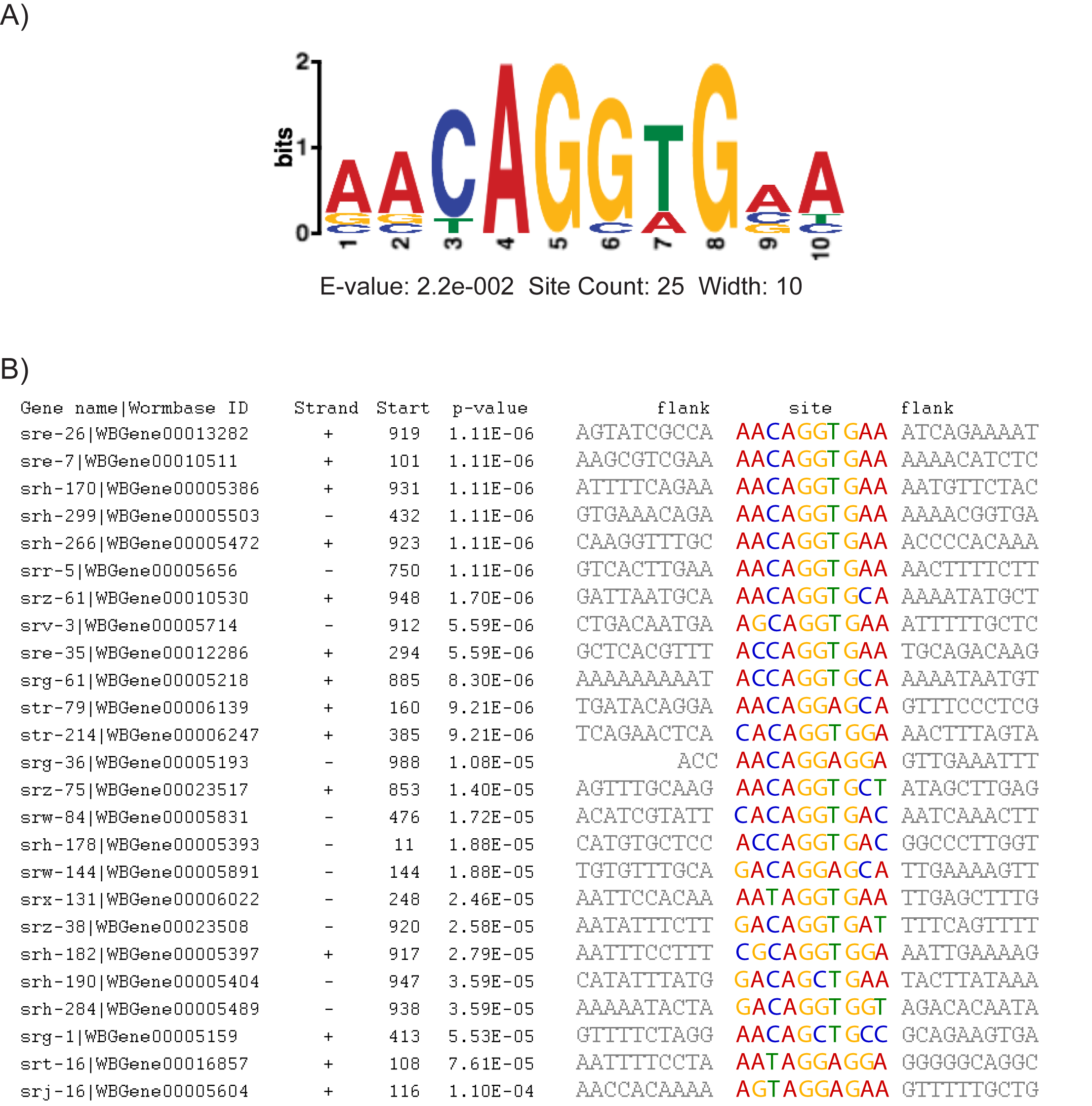

Supplement: S3 Fig — (A) E-box schematic for the serpentine chemoreceptors as modified from the MEME output [107,108]. (B) List of E-box elements in the promoters of the serpentine chemoreceptors as modified from the MEME output [107,108]. (TIF) [file pgen.1006469.s003.tif]

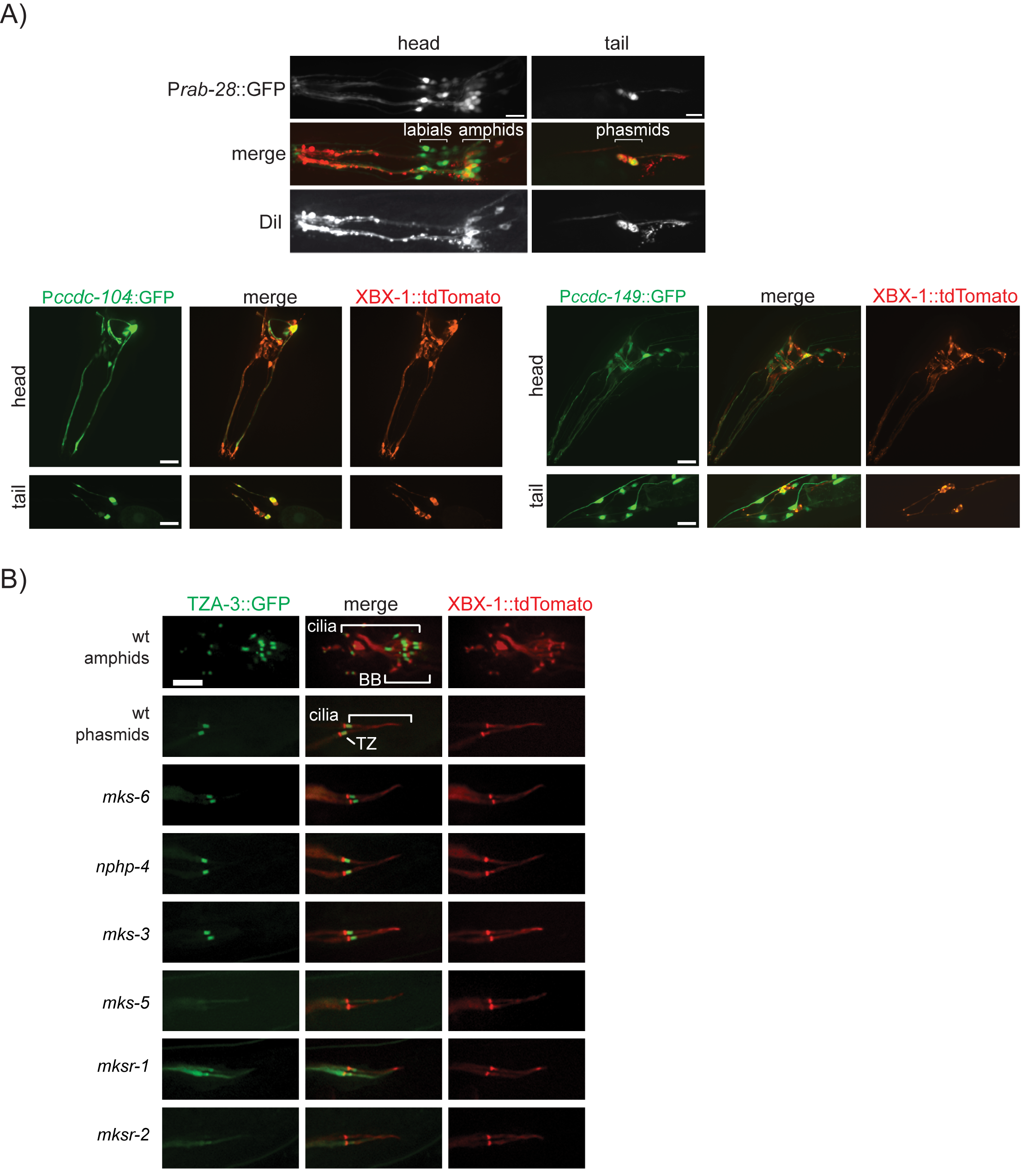

Supplement: S4 Fig — (A) Representative head and tail images of worms expressing transcriptional GFP reporters under the control of the indicated gene promoter (P). Worms expressing Prab-28::GFP were co-stained with DiI to label 6 pairs of amphid (head) ciliated neurons and the pair of phasmid (tail) ciliated neurons. Worms with Pccdc-104::GFP or Pccdc-149::GFP were co-expressed with XBX-1::tdTomato to identify ciliated cells. Scale bars; 15 μm. (B) Localisation of a translational TZA-3::GFP reporter with the ciliary protein XBX-1::tdTomato. TZA-3 localises to the transition zone (TZ) in N2 wild type (wt) amphids and phasmids. TZA-3 remains at the TZ in mks-3, mks-6 and nphp-4 mutants. However, strong localisation to the TZ is not observed in mksr-1 and mksr-2 mutants, and loss of TZA-3 ciliary base localisation is seen in the mks-5 mutant. Scale bar; 5 μm. (TIF) [file pgen.1006469.s004.tif]

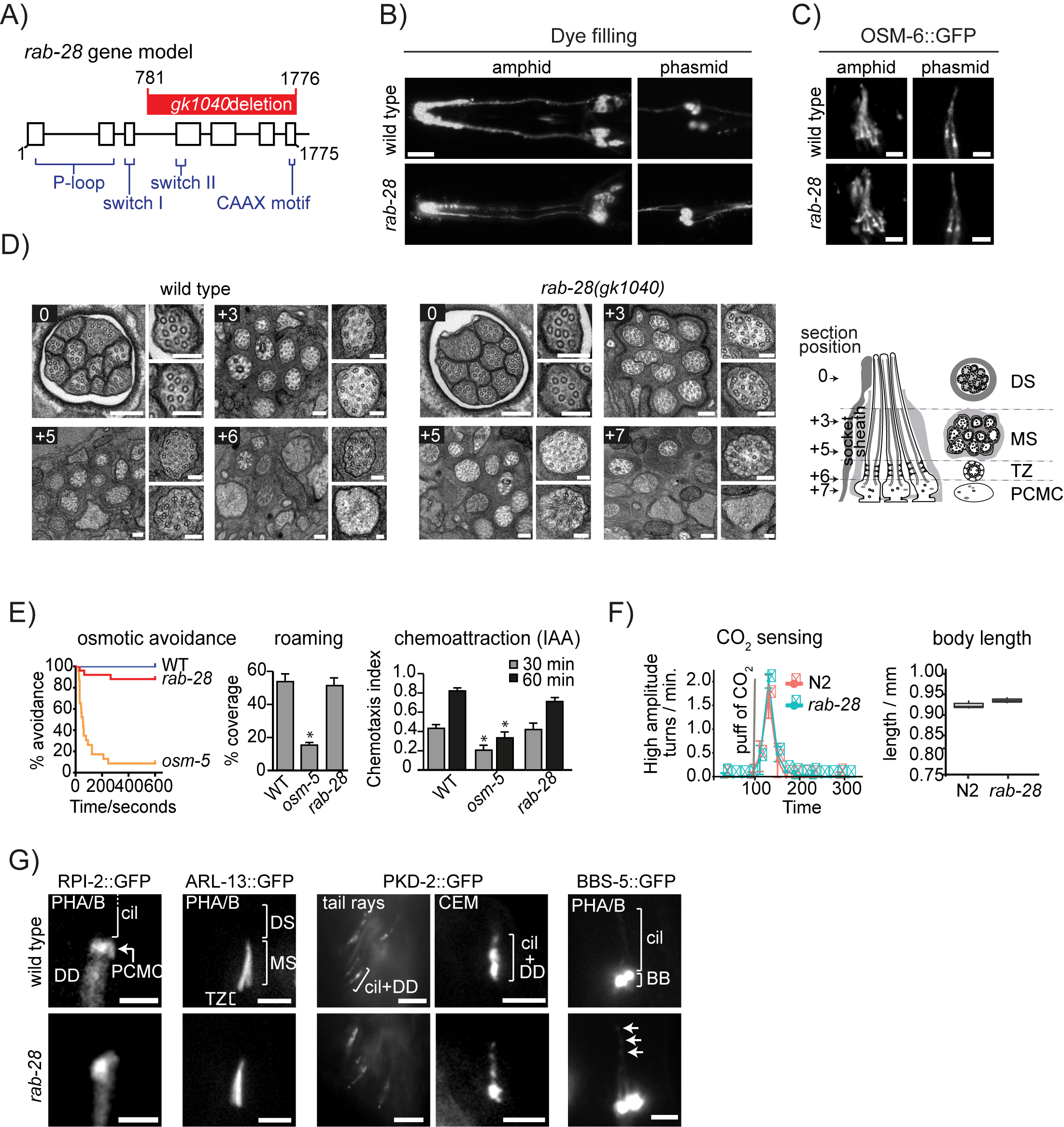

Supplement: S5 Fig — (A) Schematic of the rab-28 gene model and location of the gk1040 deletion. Exons denoted by boxes. Numbering refers to genomic nucleotide positions from the start codon in exon 1. Deletion breakpoints (781–1776) determined via Sanger sequencing. gk1040 removes the critical GTP-binding switch II domain and the CAAX box essential for RAB protein membrane association. (B) Representative images of the head and tail regions of N2 wild type and rab-28(gk1040) mutant worms following a DiI incorporation assay into amphid and phasmid neurons. Scale bar; 20 μm. (C) Representative images of amphid and phasmid cilia from N2 wild type and rab-28(gk1040) worms expressing OSM-6::GFP. Scale bars; 2 μm. (D) Transmission electron microscopy images of the amphid pore from serial cross sections of N2 wild type and rab-28(gk1040) worms. Low (large panels) and high (small panels) magnification images are shown. Images representative of at least 4 analysed pores for each strain. Both worms show 10 ciliary axonemes in the amphid pore, with each axoneme consisting of a distal segment (DS), middle segment (MS), transition zone (TZ) and periciliary membrane compartment (PCMC). Cartoon shows the amphid channel in cross section and longitudinal orientations (only 3 of the 10 axonemes shown for simplicity in longitudinal cartoon). Numbers above images indicate the position of the section relative to the most anterior section (at ‘0’); section positions also indicated in cartoon. Scale bars; 200 nm (large panels); 100 nm (small panels). (E) Assessment of rab-28(gk1040) cilia-related sensory behaviours. Shown is a population-based isoamyl alcohol (IAA) attraction assay (n = 8 for N2 wild type and osm-5; n = 12 for rab-28), as well as single worm assays that measure roaming (n = 35 for all strains) and osmotic avoidance (n = 86 for N2; n = 37 for osm-5; n = 70 for rab-28) behaviours. % avoidance (fraction of worms that avoid -do not cross—the osmotic barrier) is plotted over the time course of the [file pgen.1006469.s005.tif]

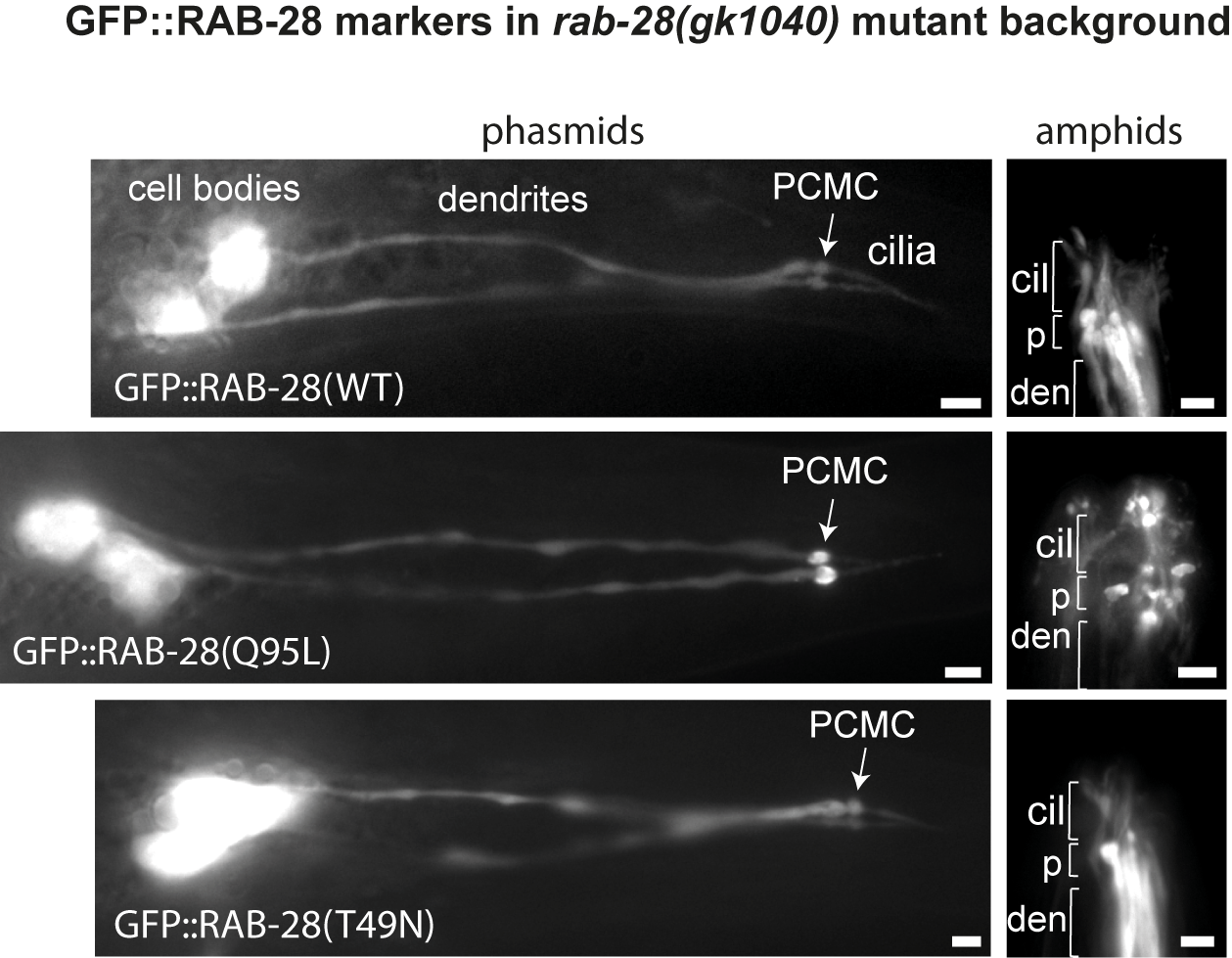

Supplement: S6 Fig — Representative images of entire phasmid tail neurons and the ciliary region of amphid head neurons from rab-28(gk1040) worms expressing GFP-tagged RAB-28(WT), RAB-28(GDP) or RAB-28(GTP). Like in a wild type background (Fig 3), all three markers are found in the cilium (cil), with RAB-28(GTP) highly enriched at the periciliary membrane. PCMC; periciliary membrane compartment (p). Den; dendrite Scale bars; 3 μm. (TIF) [file pgen.1006469.s006.tif]

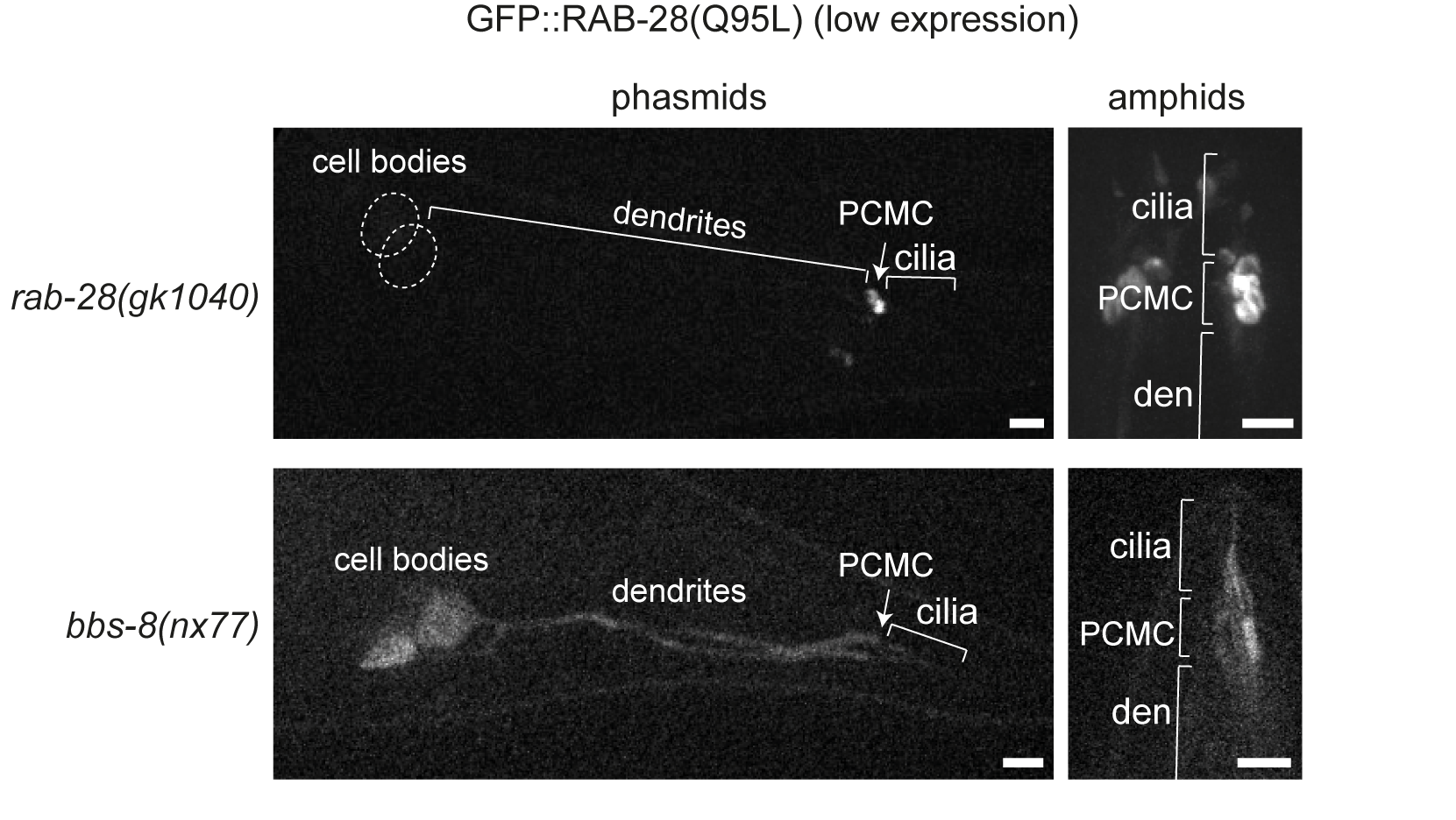

Supplement: S7 Fig — Representative images of whole phasmid neurons and the ciliary region of amphid neurons from worms with an GFP::RAB-28(GTP) transgene (oqEx304) expressed at low levels. In the rab-28(gk1040) background, RAB-28(GTP) localises exclusively to the periciliary membrane, with weaker signals in the ciliary axoneme. In bbs-8(nx77) worms, no periciliary membrane enrichment is observed for RAB-28(GTP). Note that the bbs-8 image has been overexposed to show the diffuse (non-specific) localisation of the faint RAB-28(GTP) signals. PCMC; periciliary membrane compartment. Den; dendrite. Scale bars; 3 um. (TIF) [file pgen.1006469.s007.tif]

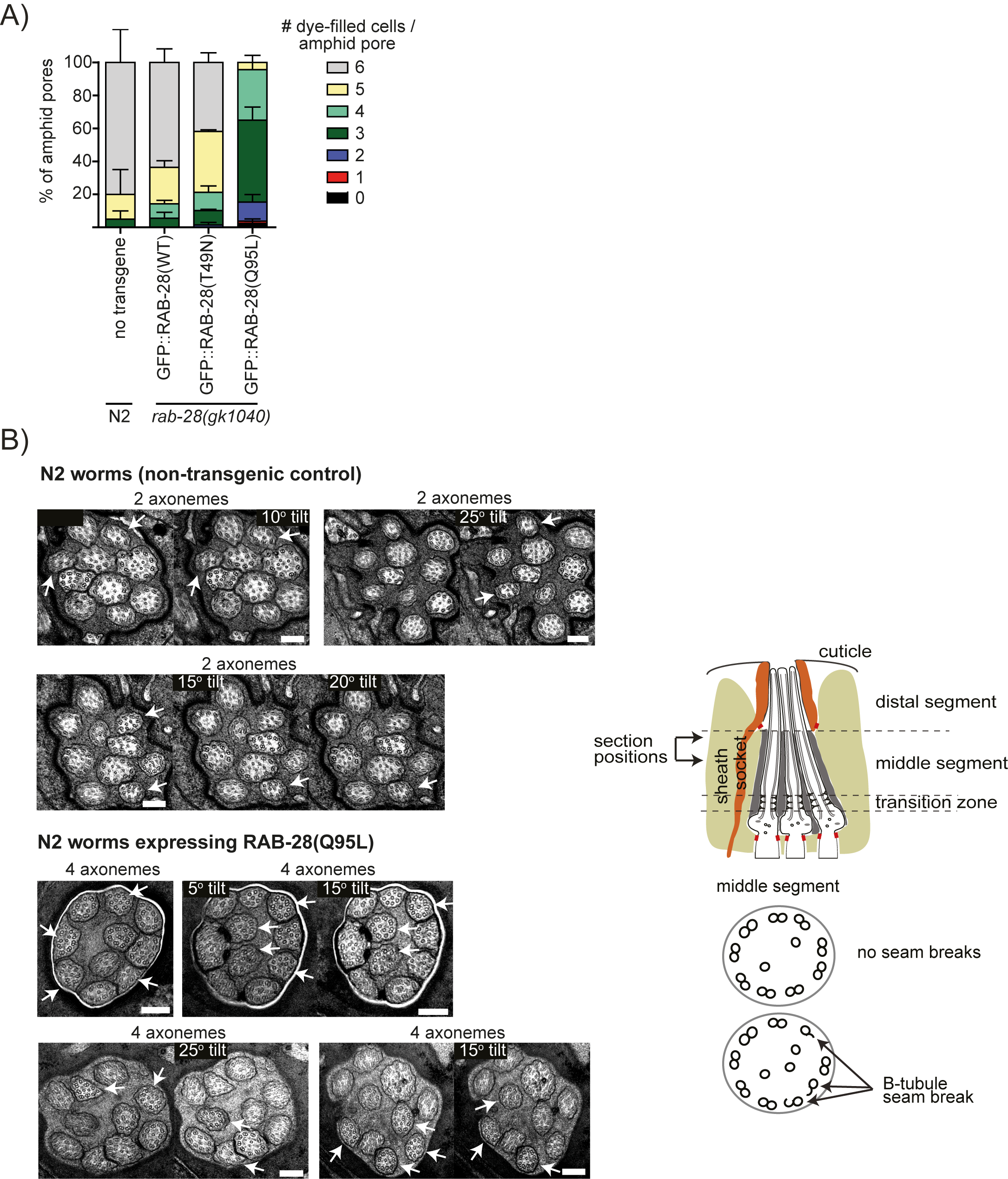

Supplement: S8 Fig — (A) Dye filling of rab-28(gk1040) worms expressing the indicated GFP-tagged RAB-28 variant (WT, GDP-locked, GTP-locked). Non-transgenic N2 worms shown as a control for the dye-filling assay. For each amphid pore, the number of dye-filling neurons was scored. Each dataset represents mean ± standard deviation (error bars) from 3 independent experiments. At least 10 (N2) or 21 (all three RAB-28 variants) amphid pores scored for each strain per experiment. (B) Transmission electron microscopy images of the amphid pore from serial cross-sections showing an increased number of B-tubule seam breaks in the middle segments of age-matched (day 1 adult) wild type (N2) and N2 worms expressing GFP-tagged RAB-28(GTP). Shown are cross sections of the distal portions of the middle segment (section positions indicated in cartoon), where the B-tubule seam break phenotype occurs. For some of the sections, out of focus ultrastructure was obtained by tilting the sections as indicated. In wild type worms, B-tubule seam breaks are observed in 2 or possibly 3 axonemes (ADL, ASI [112]), whereas in worms expressing RAB-28(GTP), B-tubule seam breaks occur in at least 4–5 axonemes. Arrows indicate axonemes with clearly identifiable b-tubule seam breaks. Cartoon shows the amphid channel in longitudinal orientation (only 3 of the 10 axonemes shown for simplicity), and indicate observed phenotypes for the distal region of the middle segment. Scale bars; 200 nm. (TIF) [file pgen.1006469.s008.tif]

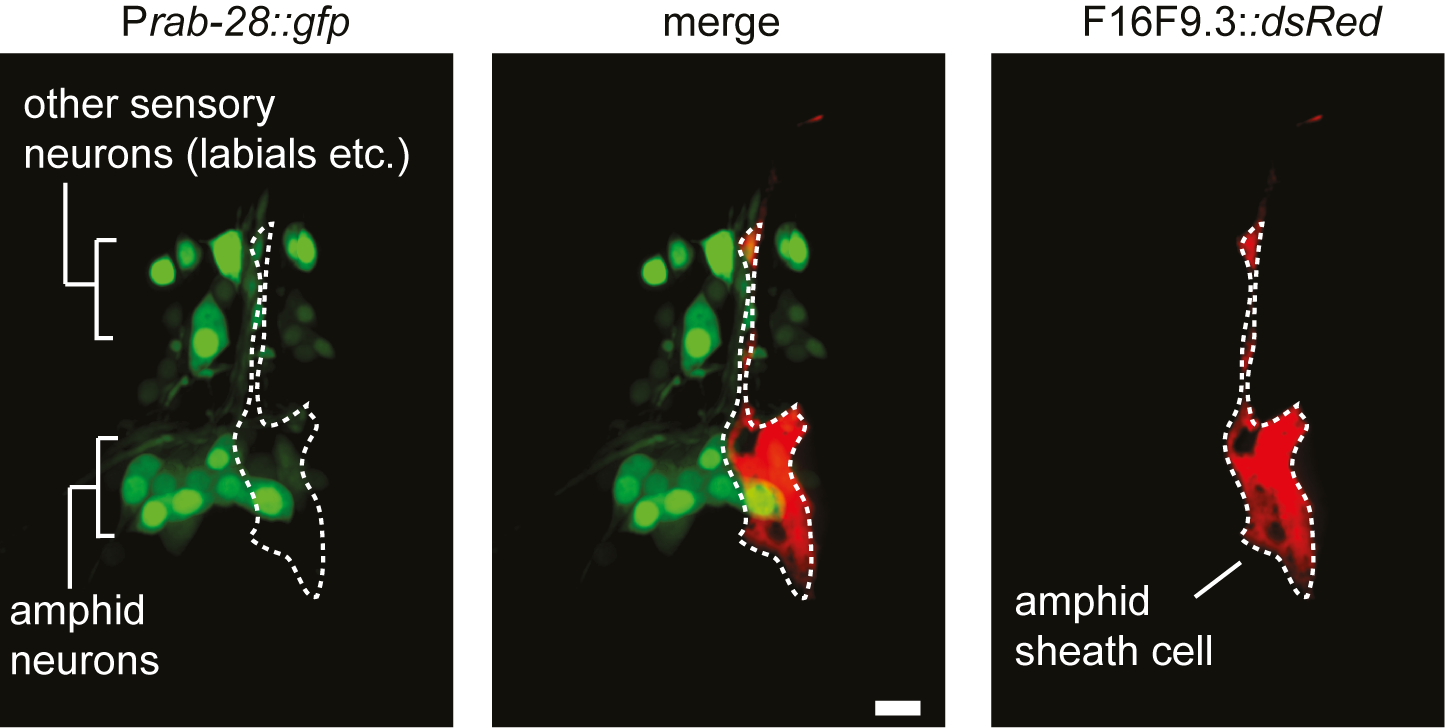

Supplement: S9 Fig — Representative images from the amphid cell body region of worms co-expressing Prab-28::gfp (GFP under the control of the rab-28 promoter; see also S4A Fig) and F16F9.3::dsRed, which is expressed exclusively in the amphid sheath cell [42]. Images show that GFP expression is restricted to ciliated neurons and does not include the sheath cell (outlined by white dotted line). This indicates that RAB-28 is not expressed in the amphid sheath cell. Scale bar; 15μm. (TIF) [file pgen.1006469.s009.tif]

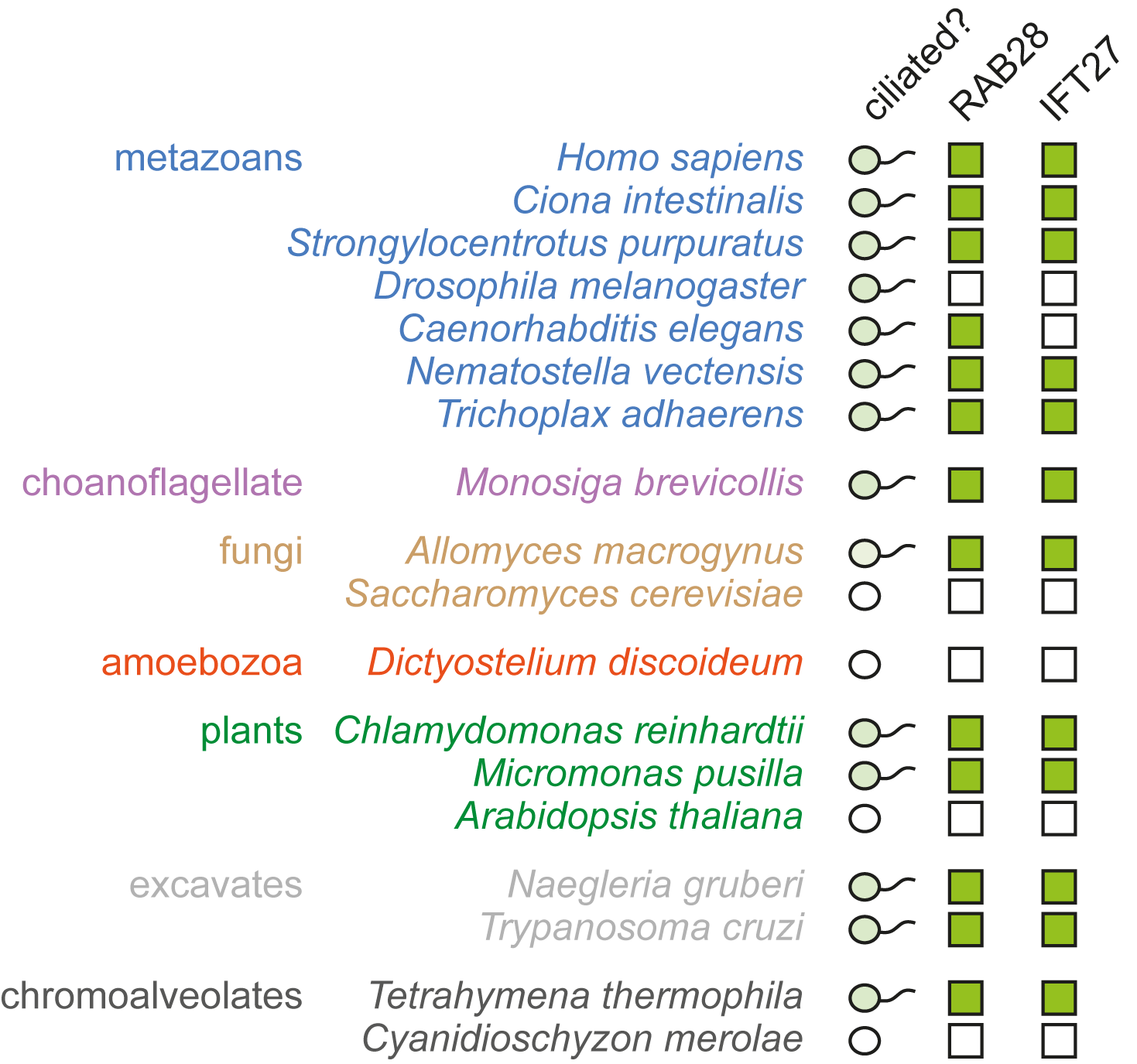

Supplement: S10 Fig — RAB28 and IFT27 protein orthologues are only found in eukaryotes that are ciliated during at least one life stage (ciliated cells are shown in green, non-ciliated cells in white). Green and white squares, respectively, denote the presence or absence of RAB28 or IFT27 in a given representative species (as presented in Elias et al. [95]). (TIF) [file pgen.1006469.s010.tif]
